# Supplementary material for: Herbivore community function shapes resilience and bistability of coral reefs
Source: PLoS Comput Biol. 2025 Jul 3;21(7):e1013221. doi: 10.1371/journal.pcbi.1013221 (PMC12240326; doi:10.1371/journal.pcbi.1013221)
Supplement: S1 Appendix — (PDF) [file pcbi.1013221.s001.pdf]

Supplement to

Herbivore community function shapes resilience and bistability of coral reefs

Shayna A. Sura[1,*,#a], James O. Lloyd-Smith[1,¶], Peggy Fong[1,¶]

[1] Department of Ecology and Evolutionary Biology, University of California Los Angeles, Los Angeles, California, USA

[#a] Current Address: National Center for Ecological Analysis and Synthesis, University of California Santa Barbara, Santa Barbara, California, USA

[*]Corresponding Author: sura@nceas.ucsb.edu (SAS)

[¶] JOL-S and PF are Joint Senior Authors.

Table of Contents

Methods **.......... 4**

van de Leemput et al (2016) Model Structure ('basic' model) 4

Recreating Figure 3e (shows hysteresis) from van de Leemput et al. (2016) ('basic' model) 4

Testing for bistability and hysteresis of van de Leemput et al. (2016) model ('basic' model) 5

Rationale for our model parameter values 5

Supplemental Question 1 7

Testing for bistability and hysteresis of our model that collapses down to the van de Leemput et al. (2016) model ('collapsed' model) 7

Sensitivity Analyses of Parameter Values I – Steady State Behavior 7

Sensitivity Analyses of Parameter Values II – Bistability 8

Results **.......... 8**

Recreation of van de Leemput et al. (2016) model ('basic' model) 8

Supplemental Question 1 9

Question 3 9

Sensitivity Analyses of Parameter Values I – Steady State Behavior 9

Sensitivity Analyses of Parameter Values II – Bistability 11

Tables **.......... 13**

Table A. 13

Equations for the 'basic' model recreated from van de Leemput et al. (2016) 13

Table B. 14

Equations for our model 14

Table C. 15

References cited by van de Leemput et al. (2016) supporting a positive feedback between coral cover and herbivore abundance. 15

Figures **.......... 16**

Figure A. 16

Diagram of the model from van de Leemput et al. (2016) 16

Figure B. 17

Hysteresis plot from van de Leemput et al. (2016) 17

Figure C. 18

Comparisons of 'basic model' and 'collapsed model'. 18
Figure D........ 19
Model output over time for varying fishing pressure and herbivore community scenarios. 19
Figure E. 20
Q1 final coral cover full heatmap results. 20
Figure F. 21
Output of our model over time for scenarios examined in Question 1. 21
Figure G........ 22
Q2 final coral cover full heatmap results. 22
Figure H........ 23
Q2 final macroalgal and turf cover full heatmap results. 23
Figure I. 24
Bistability and hysteresis plots for our 'collapsed' model. 24
Figure J. 25
Bistability plots for different B0 (initial abundances of browsers). 25
Figure K........ 26
Hysteresis plot for B0 = 0.7. 26
Figure L. 27
Supplemental Question 1 final coral cover heatmap results. 27
Figure M. 28
Sensitivity analysis for competitive effects of macroalgae and turf on coral (parameters α_M and α_T). 28
Figure N........ 29
Sensitivity analysis for mortality of macroalgae and turf from herbivory (parameters g_M and g_T). 29
Figure O........ 30
Sensitivity analysis for import of coral and turf propagules (parameters i_C and i_T). 30
Figure P. 31
Sensitivity analysis for import of coral and turf propagules (parameters i_C and i_T). 31
Figure Q........ 32
Sensitivity analysis for transition from turf to macroalgae (parameter gamma γ). 32
Figure R. 33
Sensitivity analysis for expansion of turf and macroalgae (parameters b_T and b_M). 33
Figure S. 34
Bistability sensitivity analysis for competitive effects of macroalgae and turf on coral (parameters α_M and α_T) in browser-dominated reefs. 34
Figure T. 35
Bistability sensitivity analysis for competitive effects of macroalgae and turf on coral (parameters α_M and α_T) for grazer-dominated reefs. 35
Figure U........ 36
Bistability sensitivity analysis for mortality of macroalgae and turf from herbivory (parameters g_M and g_T) for browser-dominated reefs. 36
Figure V........ 37
Bistability sensitivity analysis for mortality of macroalgae and turf from herbivory (parameters g_M and g_T) for grazer-dominated reefs. 37
Figure W. 38
Bistability sensitivity analysis for import of coral and turf propagules (parameters i_C and i_T) for browser-dominated reefs. 38
Figure X........ 39
Bistability sensitivity analysis for import of coral and turf propagules (parameters i_C and i_T) for grazer-dominated reefs. 39
Figure Y........ 40
Bistability sensitivity analysis for expansion of turf and macroalgae (parameters b_T and b_M) for browser-dominated reefs. 40

Figure Z. 41
Bistability sensitivity analysis for expansion of turf and macroalgae (parameters b_T and b_M) for grazer-dominated reefs. *41*
Figure AA. 42
Bistability sensitivity analysis for transition from turf to macroalgae (parameter gamma γ) for browser-dominated reefs. *42*
Figure BB. 43
Bistability sensitivity analysis for transition from turf to macroalgae (parameter gamma γ) for grazer-dominated reefs. *43*

Methods

van de Leemput et al (2016) Model Structure ('basic' model)

We recreated a portion of the model from van de Leemput et al. (2016), which has coral, herbivores, and algae as three state variables (Fig. A, Table A). Their model is continuous time and deterministic. Coral, algae, and herbivores all have density-dependent negative feedbacks to prevent unlimited population growth. The basic model also incorporated three de-stabilizing positive feedbacks: 1) decreased herbivory rate with increased algal cover via a Holling type II functional response for the herbivore, 2) negative effects of algal cover on coral recruitment and growth, and 3) increased herbivore carrying capacity with increased coral cover.

In their basic model, benthic cover of each state variable is modeled as the proportion of space occupied, with coral (C) and algae (A) competing for benthic space, and unoccupied space (S) not covered by coral or algae.

(1) $$S = 1 - C - A$$

Both coral (C) and algal (A) cover expand into unoccupied space through import of propagules (i_C, i_A) and expansion of existing cover (b_C, b_A). Expansion of coral cover is negatively affected by the competitive effect of algae (α_A) on coral recruitment and growth, which is proportional to the cover of algae. Coral cover decreases via a constant decay rate (d_C) representing coral mortality. Herbivores negatively affect algae via consumption, but there is a positive feedback between algal cover and herbivory rate, which is incorporated as the Holling type II functional response. Thus, grazing rate (g_A) saturates with increasing algal cover and is affected by the handling time of algae for herbivores (η_A).

(2) $$\frac{dC}{dt} = (i_C + b_C C)S(1 - \alpha_A A) - d_C C$$

(3) $$\frac{dA}{dt} = (i_A + b_A A)S - \frac{g_A A H}{g_A \eta_A A + 1}$$

Herbivores (H) are modeled as a proportion of herbivore carrying capacity. Herbivores increase based upon a growth rate (r) and decrease based upon fishing pressure (*f*). Coral has a positive effect on herbivores by providing habitat and shelter; therefore, herbivore carrying capacity is affected by coral cover based upon the parameter σ. As σ increases, coral cover increasingly influences herbivore carrying capacity.

(4) $$\frac{dH}{dt} = rH(1 - \frac{H}{(1 - \sigma) + \sigma C}) - fH$$

Recreating Figure 3e (shows hysteresis) from van de Leemput et al. (2016) ('basic' model)

We used the model equations, initial conditions, and parameter values (Table A) from van de Leemput et al. (2016) to reconstruct their model ('basic' model). We tested our model by

replicating their Figure 3e. To recreate this figure, we examined the model for hysteresis by using simulations of increasing and decreasing fishing pressure and determining the tipping points where the coral reef system switches from one stable state to another. Specifically, for the "forward" simulations, we increased fishing pressure from 0 – 1 in 0.005 increments, and for the "reverse" simulations, we decreased fishing pressure from 1 – 0 in 0.005 increments. We ran each simulation for 1000 years to ensure the system reached equilibrium (hereafter stated as steady state). For each fishing pressure, we used the end conditions for the state variables from the previous fishing pressure as the initial conditions for the next fishing pressure.

Testing for bistability and hysteresis of van de Leemput et al. (2016) model ('basic' model)

Because we are also interested in bistability, we wanted to determine what levels of fishing pressure cause bistability in the basic model. Bistability in our model is indicated by the final coral cover being split between high (>0.6) and low (<0.2) coral cover equilibrium states for the same level of fishing pressure, but dependent upon initial coral cover conditions. To test for bistability, we ran the model for 1000 years (to steady state) with 100 different initial coral covers (0 – 1 in increments of 0.01) and 100 different fishing pressures (0 – 1 in increments of 0.01), using the initial conditions and parameter values from van de Leemput et al. (2016) and listed in Table A.

Rationale for our model parameter values

Our 5 Assumptions:

1. No handling time for turf algae ($\eta_T = 0$).

Rationale: We assume no handling time for turf algae because turf algae are easily consumed since they lack physical and chemical defenses, commonly found in macroalgae, that reduce herbivory rates (Littler et al. 1983, Steneck and Dethier 1994).

2. Expansion of macroalgae is slower than expansion of turf algae ($b_M < b_T$).

Rationale: We assume expansion is slower for macroalgae compared to turf algae because turf algae are highly productive (Carpenter 1985, Hatcher 1988, Klumpp and McKinnon 1992) and typically have higher productivity than macroalgae (e.g., Littler et al. 1983, Steneck and Dethier 1994). Also, McClanahan et al. (2002) found that turf algae expanded to much higher percent cover over a 50-day period compared to macroalgae, regardless of experimental treatment, which included a caged control.

We kept $b_T = 0.8$ to correspond with the b_A value from the van de Leemput et al. (2016) model. For the main text, we lowered b_M to 0.5 based upon our assumption that it should be lower than b_T, but acknowledge this reduction was based on qualitative rather than quantitative empirical support. Thus we did a sensitivity analysis examining choices for b_T and b_M values ranging from 0.1 – 0.9 under varying fishing pressures (0.1 – 0.7) and herbivore community scenarios (no

generalists, even herbivore community, browser dominated, grazer dominated, and generalist dominated). The results of the sensitivity analysis are presented in Figure R.

3. Macroalgae grows from recruits present in turf ($i_M = 0$).

Rationale: We assume there is no importation of macroalgal propagules because we define turf algae as sparse to thick mats containing an assemblage of fast-growing filamentous algae and juvenile macroalgae that are cropped short by herbivores (Littler and Littler 2011a). Thus, macroalgae, which are an assemblage of erect, fleshy algae typically > 2 cm tall (Littler and Littler 2011b), grow from turf algae as represented in our model.

4. Mortality due to herbivory is greater for turf than macroalgae ($g_T > g_M$).

Note: In our model, mortality of turf and macroalgae refers to reductions in their benthic cover because their populations are modeled as proportions of benthic space.

Rationale: We assume higher mortality due to herbivory for turf algae compared to macroalgae because macroalgae often have chemical and/or physical defenses, which reduce herbivory (Fong and Paul 2011). Functional form models also predict that turf algae are more susceptible to herbivory than macroalgae (Littler et al. 1983, Steneck and Dethier 1994).

We kept $\mathbf{g}_M = 1$ to correspond with the $\mathbf{g}_A$ value from the van de Leemput et al. (2016) model. We increased $\mathbf{g}_T$ to 2 based upon our assumption that it should be higher than $\mathbf{g}_M$. As choice of an increase was supported by qualitative not quantitative data, we did a sensitivity analysis examining for $\mathbf{g}_T$ and $\mathbf{g}_M$ values ranging from 0 – 8 under varying fishing pressures (0.1 – 0.7) and herbivore community scenarios (no generalists, even herbivore community, browser dominated, grazer dominated, and generalist dominated). The results of the sensitivity analysis are presented in Figure N.

5. Macroalgae exert a stronger competitive effect on coral than turf ($\alpha_M > \alpha_T$).

Rationale: We make this assumption because the settlement of coral larvae may or may not be reduced by turf algae, depending on the type of turf and presence or absence of sediment trapped by the turf (Fong and Paul 2011). In contrast, macroalgae is predominantly thought to inhibit settlement and recruitment of coral larvae (McCook et al. 2001, Fong and Paul 2011, Schmitt et al. 2022). Furthermore, macroalgae have more mechanisms than turf (filamentous) algae to out-compete juvenile and adult coral, including allelopathy, overgrowth, shading, and abrasion (McCook et al. 2001, Cheh et al. 2024).

We kept $\boldsymbol{\alpha}_M = 0.5$ to correspond with the $\boldsymbol{\alpha}_A$ value from the van de Leemput et al. (2016) model. We decreased $\boldsymbol{\alpha}_T$ to 0.25 based upon our assumption that it should be lower than $\boldsymbol{\alpha}_M$. Because our value selection was based on qualitative instead of quantitative data, we did a sensitivity analysis examining for $\boldsymbol{\alpha}_T$ and $\boldsymbol{\alpha}_M$ values ranging from 0.1 – 0.9 under varying fishing pressures (0.1 – 0.7) and herbivore community scenarios (no generalists, even herbivore community, browser dominated, grazer dominated, and generalist dominated). The results of the sensitivity analysis are presented in Figure M.

Supplemental Question 1

Supplemental Question 1: *How does varying initial benthic cover of coral, turf, and macroalgae influence the recovery of coral reefs after a disturbance, for different levels of fishing pressure?*

Disturbances can result in drastically different reductions of coral cover (Edmunds et al. 2019). To explore how this might influence reef recovery, we set initial coral cover to 0.15, 0.35, 0.55, and 0.75. For each initial coral cover, we varied initial cover of turf and macroalgae from 0 – 0.7, or up to the appropriate level given that we held initial unoccupied space (S) to a minimum of 0.15 for all simulations. Therefore, we constrained the total initial benthic cover (turf + macroalgae + coral) to ≤0.85. We set fishing pressure to 0 – 0.7 in increments of 0.1.

Testing for bistability and hysteresis of our model that collapses down to the van de Leemput et al. (2016) model ('collapsed' model)

As a baseline for our expanded model, we examined both bistability and hysteresis for our model using the parameter values and initial conditions that collapse to the van de Leemput et al (2016) model, which we refer to as our 'collapsed' model.

We examined bistability as stated above. To examine the model for hysteresis, we did "forward" and "reverse" simulations, reflecting increasing or decreasing fishing pressure, respectively. Then we determined whether transitions from high to low coral cover states differed based on direction of change in this environmental driver. Specifically, for the "forward" and "reverse" simulations, we increased then decreased fishing pressure from 0 – 1 in 0.005 increments, respectively. For each simulation, we used the end conditions for the state variables from the previous simulation for the next simulation.

Sensitivity Analyses of Parameter Values I – Steady State Behavior

We did sensitivity analyses to explore how some of our selected parameter values may influence the steady state behavior of our model. Specifically, we did the following sensitivity analyses:

1) Competitive effects of macroalgae and turf on coral (parameters α_M and α_T) for values ranging from 0.1 – 0.9 and under varying fishing pressure conditions (0.1 – 0.7) and herbivore community scenarios (no generalists, even herbivore community, browser dominated, grazer dominated, and generalist dominated). We focused on combinations of parameter values that were consistent with our qualitative assumptions (e.g., we focused on combinations $\alpha_M > \alpha_T$).

2) Mortality rates of macroalgae and turf from herbivory (parameters g_M and g_T) for values ranging from 0 – 8 and under varying fishing pressures (same as above) and herbivore community scenarios (same as above). We focused on combinations that met our assumption of $g_T > g_M$.

3) Import of coral and turf propagules (parameters i_C and i_T) for values ranging from 0 – 0.3 and under varying fishing pressure conditions (same as above) and herbivore community scenarios (same as above). We also explored i_C and i_T values ranging from 0 – 0.3 when starting

with different initial coral cover conditions (initial coral = 0.75, 0.55, 0.35, and 0.15) under varying fishing pressures (same as above).

4) Transition from turf to macroalgae (parameter gamma γ) for values ranging from 0 – 0.9, and we explored these values in relation to initial turf cover (T0 values from 0 – 0.85), and under varying fishing pressures and herbivore community scenarios as above.

5) Expansion of turf and macroalgae (parameters b_T and b_M) for values ranging from 0.1 – 0.9 under varying fishing pressures and herbivore community scenarios as above. We focused on combinations that met our assumption of $b_M < b_T$.

Sensitivity Analyses of Parameter Values II – Bistability

We also did sensitivity analyses to explore how these same parameters may influence the occurrence of bistability under browser-dominated and grazer-dominated scenarios. We focused on these two herbivore community scenarios since these are the ones under which our model demonstrates lack of bistability (for browser-dominated community) or continued bistability with complete reduction of fishing pressure (for grazer-dominated community). We conducted our bistability analyses the same as for our main text analyses, except (due to computational time limitations) we examined combinations of initial coral cover and fishing pressure values in increments of 0.02 (compared to 0.01 increments for main text analyses). Based upon the output from the previous sensitivity analyses, we examined a smaller range of parameter values from above:

1) α_M and α_T values ranging from 0.1 – 0.7 in increments of 0.1. We focused on combinations that met our assumption of $\alpha_M > \alpha_T$.

2) g_M and g_T values ranging from 0 – 6 in increments of 1. We focused on combinations that met our assumption of $g_T > g_M$.

3) i_C and i_T values ranging from 0 – 0.3 in increments of 0.05.

4) b_T and b_M values ranging from 0.3 – 0.9 in increments of 0.1. We focused on combinations that met our assumption of $b_M < b_T$.

5) Gamma (γ) values ranging from 0 – 0.6 in increments of 0.05.

Results

Recreation of van de Leemput et al. (2016) model ('basic' model)

We adequately reconstructed the van de Leemput et al. (2016) model in R, as shown by our recreation model exhibiting hysteresis for the same fishing pressures as those from Figure 3e in van de Leemput et al. (2016) (Fig. Bi-ii). Our recreation of the van de Leemput et al. (2016) model exhibits bistability, with final coral cover dependent on initial coral cover, for fishing pressures between 0.33 – 0.51 (Fig. Biii).

Supplemental Question 1

How does varying initial benthic cover of coral, turf, and macroalgae influence the recovery of coral reefs after a disturbance, for different levels of fishing pressure?

In contrast to the impacts of herbivore community composition on coral reef recovery, initial cover of turf and macroalgae have relatively little effect on reef recovery. Coral reef recovery is less likely on highly disturbed reefs (Fig. L, left column). Fishing pressure has less of a negative effect on reef recovery as initial coral cover increases (Fig. L compare columns from left to right). The relative initial cover of turf versus macroalgae only affects reef recovery when fishing pressure is intermediate (0.5) and initial coral cover is low (≤0.35, Fig. L). Overall, for scenarios with lower initial coral cover and higher fishing pressure, coral reefs can recover better when there is higher initial cover of turf compared to macroalgae (Fig. L panel in first column and sixth row, light band along x-axis).

Question 3

How does variation in the dominant herbivore functional group influence the occurrence of alternative stable states in response to fishing pressure?

As a baseline, we examined bistability and hysteresis for our model using the parameter values and initial conditions that collapse to the van de Leemput et al (2016) model. Results are shown in Figure Ii-ii. When initial herbivore abundances are even, our full model exhibits bistability for a very similar, although slightly contracted, range of fishing pressures compared to our 'collpased' model that corresponds to the van de Leemput et al. (2016) model (main text Fig. 6A vs. Fig. Ii). The collapsed model (Fig. Iii) and 'even herbivore community' scenario (main text Fig. 6E) show similar hysteresis curves, with the latter having a slightly contracted range of hysteresis.

Sensitivity Analyses of Parameter Values I – Steady State Behavior

To interpret the results of the sensitivity analyses of our parameter values, one outcome of interest is the slope of the boundary line between regions of parameter space that lead to steady states of low versus high coral cover. Specifically, the angle of the boundary line informs the relative sensitivity of the system's steady state to changes in parameter values. For example, for our $\boldsymbol{\alpha_M}$ and $\boldsymbol{\alpha_T}$ parameters, we have $\boldsymbol{\alpha_T}$ on the x-axis and $\boldsymbol{\alpha_M}$ on the y-axis (Fig. M). If the boundary line was **horizontal**, that would indicate that the value of $\boldsymbol{\alpha_T}$ can change from 0 to 0.9 and there would be no change in whether the reef steady state is high or low coral cover. Thus, a horizontal line indicates the model scenario is completely insensitive to changes in $\boldsymbol{\alpha_T}$, but is sensitive to changes in $\boldsymbol{\alpha_M}$. Conversely, a **vertical boundary line** indicates the opposite; the system's steady state is completely insensitive to changes in $\boldsymbol{\alpha_M}$, but is sensitive to changes in $\boldsymbol{\alpha_T}$. The two parameter values are equally important when the boundary line has a 45-degree angle (on equally scaled axes), such that any change in the value of one parameter has equal influence to a change of the same size in the second parameter.

For boundary lines that have an angle between these example cases, the relative sensitivity of the system state to the two parameter values can be determined by whether the slope of the line is closer to a horizontal or vertical orientation. Boundary lines with angles closer to horizontal (called 'low angles' below) indicate that the parameter value on the y-axis has more influence on the steady state. Boundary lines with angles closer to vertical (called 'steep angles' below) indicate that the parameter value on the x-axis has more influence on the steady state.

1) Competitive effects of macroalgae and turf on coral (parameters α_M and α_T) – Coral cover is favored by lower values of α_M and α_T, as expected, since lower competitive effects of algae (either macroalgae or turf) will benefit coral (Fig. M). The influence on coral reef steady state is stronger for α_M compared to α_T parameter (given the low angle of the boundary line between low coral vs. high coral cover states, which reflects greater sensitivity of the steady state to the value of α_M compared to α_T).This is also expected given other constraints on turf and macroalgae (e.g., higher mortality from grazing on turf versus macroalgae).

The dependence of coral reef state on the α_M and α_T parameters varies between herbivore community scenarios. These variations are consistent with expectations; for example, the influence of α_T is stronger (i.e. the angle of the boundary line is steeper) in browser-dominated scenarios, which is consistent with there being fewer herbivores exerting top-down control on the turf algae.

2) Mortality rates of macroalgae and turf from herbivory (parameters g_M and g_T) – Coral cover is favored by higher values of g_M and g_T, as expected, since higher herbivory mortality of algae (macroalgal or turf) will benefit coral (Fig. N). The influence on coral reef steady state is stronger for g_M compared to g_T (given the low angle of the boundary line between low coral vs. high coral cover states). This is also expected given other constraints on turf and macroalgae (e.g., higher competitive effect of macroalgae than turf on coral).

The dependence of coral reef state on the g_M and g_T parameters varies between herbivore community scenarios. These variations are consistent with expectations; for example, the herbivory mortality of macroalgae (g_M) must be higher under grazer-dominated scenarios for the steady state to be coral dominated. This is consistent with less top-down control on macroalgae when grazers are dominant.

3) Import of coral and turf propagules (parameters i_C and i_T) – Coral cover is favored with higher values of i_C and lower values of i_T, as expected (Fig. O, P). The influence on coral reef steady state is generally stronger for i_C compared to i_T (given the low angle of the boundary line). However, the influence of i_C and i_T on coral reef steady state also depends upon the herbivore community scenario, fishing pressure, and initial coral cover (Fig. O, P). Interestingly, the influence of i_T on coral steady state increases (i.e. the angle of the boundary line becomes steeper) as fishing pressure increases and is most evident under grazer-dominated scenarios (Fig. O). This effect arises because turf can mature into macroalgae, which can then outcompete coral if there is a paucity of top-down pressure from browsers and generalists.

4) Transition from turf to macroalgae (parameter gamma γ) – Coral cover is favored by lower values of γ, as expected, since slower transitions from turf to macroalgae will benefit coral since macroalgae have stronger competitive effects on coral (Fig. Q). The influence of γ on coral reef steady state is dependent upon herbivore community scenarios and fishing pressure,

thereby behaving as expected. For example, there is a strong influence of **γ** on coral reef steady state when grazers are dominant, since lack of top-down pressure on turf means that rapid transitions to macroalgae leads them to rapidly out-compete coral. Meanwhile, the influence of **γ** is weaker when the herbivore community is more balanced or has plenty of browsers or generalists to exert top-down control on algae once it transitions from turf to macroalgae.

5) Expansion of turf and macroalgae (parameters b_T and b_M) – Coral cover is favored by lower values of b_T and b_M, as expected, since slower expansion of algae (macroalgae or turf) will benefit coral (Fig. R). The influence on coral reef steady state is stronger for b_M compared to b_T (given the generally low angle of the boundary line between low versus high coral cover states). This is also expected given other constraints on turf and macroalgae (e.g., macroalgae has a stronger competitive effect on coral versus turf).

The influence of b_T on coral reef steady state becomes stronger under browser-dominated scenarios, specifically at higher fishing pressures, demonstrated by the high coral cover disappearing with b_T values higher than 0.6 at the highest fishing pressure (and also by the region of steep boundary line between low versus high coral states). This is consistent with browsers not exerting top-down control on turf algae, leading to more opportunities for turf to spread, mature into macroalgae, and out-compete coral.

Sensitivity Analyses of Parameter Values II – Bistability

The sensitivity analyses of how some parameter values influence the occurrence of bistability for browser-dominated and grazer-dominated scenarios can be interpreted the same as for our main text analyses and figures. Specifically, bistability is evident when, for a given fishing pressure, both low (<0.2) and high (>0.6) final coral covers occur, depending only upon the initial coral cover.

1) Competitive effects of macroalgae and turf on coral (parameters α_M and α_T) – For browser-dominated scenarios, changes in the competitive effects of macroalgae and turf on coral (α_M and α_T) have minimal impacts on the occurrence of bistability (Fig. S). Similarly, for grazer-dominated scenarios, changes in the α_M and α_T parameters have a minimal impact on the occurrence of bistability (Fig. T). However, we do see that for very low values of $\boldsymbol{\alpha_M}$ =< 0.2, coral reefs can stop exhibiting bistability and recovery to coral dominance can occur for any initial coral cover, for very low values of fishing pressure (Fig. T).

2) Mortality rates of macroalgae and turf from herbivory (parameters g_M and g_T) – For browser-dominated scenarios, changes in the mortality rates of macroalgae and turf from herbivory (g_M and g_T) impact the occurrence of bistability (Fig. U). The absence of bistability for browser-dominated scenarios, as reported in the main text, remains evident when g_M and g_T are relatively small and equal to each other (e.g., both set to 2). Bistability becomes more evident when g_T values become larger than g_M values (Fig. U). For grazer-dominated scenarios, the loss of bistability at very low fishing pressures (as reported in the main text) occurs when g_M >= 2, regardless of the value of g_T (Fig. V). Altogether, this sensitivity analysis shows that the relative strength of mortality of macroalgae and turf due to herbivory is an important driver of alternative stable states within a coral reef system.

3) Import of coral and turf propagules (parameters i_C and i_T) – For browser-dominated scenarios, changes in the import of coral and turf propagules (i_C and i_T) have minimal impacts on the occurrence of bistability (Fig. W). Specifically, changes in i_T values have no impact as long as $i_T >= 0.05$, while decreasing i_C values increase the range of bistability when $i_T = 0$ (Fig. W). For grazer-dominated scenarios, changes in i_C and i_T values have a greater impact on the range of bistability, with recovery to high coral cover states possible at low fishing pressure as i_C increases, and reduction in the strength of bistability as i_T increases (Fig. X).

4) Expansion of turf and macroalgae (parameters b_T and b_M) – For browser-dominated scenarios, changes to the expansion rate of macroalgae (b_M) influence the occurrence of bistability (Fig. Y), however, changes to the expansion rate of turf (b_T) have minimal to no effects on bistability (Fig. Y). Specifically, as b_M values increase, the coral reef system can exhibit clear bistability (Fig. Y), which was not evident for the default parameters in the main text. Similarly, for grazer-dominated scenarios, decreases in b_M values reduce the range of bistability, thus allowing recovery to coral dominance for low fishing pressure values when expansion rates of macroalgae are slower (Fig. Z). This makes sense that weakening the expansion of macroalgae would facilitate coral recovery while weakening the expansion of turf does not have as much of an impact because macroalgae has a stronger competitive effect than turf on coral.

5) Transition from turf to macroalgae (parameter gamma γ) – For browser-dominated scenarios, increasing the value of the parameter for the transition from turf to macroalgae (γ) results in bistability occurring (Fig. AA). For grazer-dominated scenarios, increasing gamma values reduce the region of bistability, but it still exists even at low fishing pressures (Fig. BB).

Overall, the sensitivity analyses indicate that some of these parameter values, especially those related to macroalgae and turf, can influence the steady state behavior and occurrence of bistability for coral reef systems. Additional research will be helpful to determine these parameter values more generally and for specific coral reef systems.

Tables

Table A. Equations for the 'basic' model recreated from van de Leemput et al. (2016), with the state variables and parameters listed below.

$S = 1 - C - A$	$\frac{dA}{dt} = (i_A + b_A A)S - \frac{g_A AH}{g_A \eta_A A + 1}$
$\frac{dC}{dt} = (i_C + b_C C)S(1 - \alpha_A A) - d_C C$	$\frac{dH}{dt} = rH(1 - \frac{H}{(1-\sigma) + \sigma C}) - fH$
**State Variables:**	**Initial Condition Values:**
S = unoccupied space	$S_{t=0} = 0.15$
C = coral cover	$C_{t=0} = 0.75$
A = algal cover	$A_{t=0} = 0.1$
H = herbivore proportion	$H_{t=0} = 0.9$

**Parameters** *(units not specified by van de Leemput et al. 2016)*:		**Values:**	
i	importation of propagules	$i_C = 0.05$	$i_A = 0.05$
b	expansion of existing adults (which is proportional to existing cover)	$b_C = 0.3$	$b_A = 0.8$
d	mortality of coral (constant decay rate)	$d_C = 0.1$	
g	mortality of algae (constant grazing rate per herbivore)		$g_A = 1$
r	growth rate of herbivores	$r = 1$	
f	herbivore mortality (constant fishing pressure)	$f = 0.1$	
η	algal handling time of herbivores		$\eta_A = 1$
α	competition effect of algae on coral recruitment and growth		$\alpha_A = 0.5$
σ	strength of relationship between coral cover and herbivore carrying capacity	$\sigma = 0.6$	

Table B. Equations for our model, with state variables and parameters listed below, and orange-colored text indicating differences from the van de Leemput et al. (2016) model. The collapsed model parameter values allow our model to collapse down to the van de Leemput et al. (2016) model. Our model ('expanded') parameter values are based upon assumptions given in the text.

$$S = 1 - C - T - M$$

$\frac{dC}{dt} = (i_C + b_C C)S(1 - (\alpha_T T + \alpha_M M)) - d_C C$	$\frac{dG}{dt} = rG\left(1 - \frac{(G+R+B)}{(1-\sigma)+\sigma C}\right) - fG$
$\frac{dT}{dt} = (i_T + b_T T)S - \gamma T - \left(\frac{g_T TG}{g_T \eta_T T + 1} + \frac{g_T TR}{g_T \eta_T T + g_M \eta_M M + 1}\right)$	$\frac{dR}{dt} = rR\left(1 - \frac{(G+R+B)}{(1-\sigma)+\sigma C}\right) - fR$
$\frac{dM}{dt} = (i_M + b_M M)S + \gamma T - \left(\frac{g_M MB}{g_M \eta_M M + 1} + \frac{g_m MR}{g_T \eta_T T + g_M \eta_M M + 1}\right)$	$\frac{dB}{dt} = rB\left(1 - \frac{(G+R+B)}{(1-\sigma)+\sigma C}\right) - fB$

**State Variables:**			
	S = unoccupied space<br>C = coral cover	T = turf algal cover<br>M = macroalgal cover	G = grazer herbivore proportion<br>R = generalist herbivore proportion<br>B = browser herbivore proportion

Initial Condition Values (unless otherwise indicated in analyses or text)

**Collapsed Model**			**Model**		
$S_{t=0} = 0.15$<br>$C_{t=0} = 0.75$	$T_{t=0} = 0.05$<br>$M_{t=0} = 0.05$	$G_{t=0} = 0.45$<br>$R_{t=0} = 0$<br>$B_{t=0} = 0.45$	$S_{t=0} = 0.15$<br>$C_{t=0} = 0.75$	$T_{t=0} = 0.10$<br>$M_{t=0} = 0.00$	$G_{t=0} = 0.3$<br>$R_{t=0} = 0.3$<br>$B_{t=0} = 0.3$

**Parameters:**		**Collapsed Model Values**	**Model Values**
i_C	importation of coral propagules	0.05 yr^{-1}	0.05 yr^{-1}
i_T	importation of turf propagules	0.025 yr^{-1}	0.05 yr^{-1}
i_M	importation of macroalgal propagules	0.025 yr^{-1}	0 yr^{-1}
b_C	expansion of existing coral (proportional to existing cover)	0.3 yr^{-1}	0.3 yr^{-1}
b_T	expansion of existing turf (proportional to existing cover)	0.8 yr^{-1}	0.8 yr^{-1}
b_M	expansion of existing macroalgae (proportional to existing cover)	0.8 yr^{-1}	0.5 yr^{-1}
d_C	mortality of coral (constant decay rate)	0.1 yr^{-1}	0.1 yr^{-1}
g_T	mortality of turf algae (constant grazing rate per herbivore)	2 yr^{-1}	2 yr^{-1}
g_M	mortality of macroalgae (constant grazing rate per herbivore)	2 yr^{-1}	1 yr^{-1}
r	growth rate of herbivores	1 yr^{-1}	1 yr^{-1}
f	herbivore mortality (constant fishing pressure)	0.1 yr^{-1}	0.1 yr^{-1}
η_T	turf algae handling time of herbivores	1	0
η_M	macroalgae handling time of herbivores	1	1
α_T	competition effect of turf algae on coral recruitment and growth	0.5	0.25
α_M	competition effect of macroalgae on coral recruitment and growth	0.5	0.5
σ	strength of relationship between coral cover and herbivore carrying capacity	0.6	0.6
γ	transition probability from turf algae to macroalgae	0 yr^{-1}	0.1 yr^{-1}

Table C. References cited by van de Leemput et al. (2016) supporting a positive feedback between coral cover and herbivore abundance.

1	Friedlander AM, Parrish JD (1998) Habitat characteristics affecting fish assemblages on a Hawaiian coral reef. J Exp Mar Bio Ecol 224:1–30
2	Garpe K, Yahya S, Lindahl U, Öhman M (2006) Long-term effects of the 1998 coral bleaching event on reef fish assemblages. Mar Ecol Prog Ser 315:237–247
3	Graham NAJ, Wilson SK, Jennings S, Polunin NVC, Bijoux JP, Robinson J (2006) Dynamic fragility of oceanic coral reef ecosystems. Proc Natl Acad Sci U S A 103:8425–8429
4	Halford A, Cheal AJ, Ryan D, Williams DM (2004) Resilience to large-scale disturbance in coral and fish assemblages on the Great Barrier Reef. Ecology 85:1892–1905
5	Lee SC (2006) Habitat complexity and consumer-mediated positive feedbacks on a Caribbean coral reef. Oikos 112:442–447
6	Lindahl U, Öhman MC, Schelten CK (2001) The 1997/1998 mass mortality of corals: effects on fish communities on a Tanzanian coral reef. Mar Pollut Bull 42:127–131
7	Mumby PJ, Wabnitz CCC (2002) Spatial patterns of aggression, territory size, and harem size in five sympatric Caribbean parrotfish species. Environ Biol Fish 63:265–279
8	Öhman MC, Munday PL, Jones GP, Caley MJ (1998) Settlement strategies and distribution patterns of coral-reef fishes. J Exp Mar Bio Ecol 225:219–238
9	Paddack MJ, Reynolds JD, Aguilar C, Appeldoorn RS, Beets J, Burkett EW, Chittaro PM, Clarke K, Esteves R, Fonseca AC, Forrester GE, Friedlander AM, García-Sais J, González-Sansón G, Jordan LKB, McClellan DB, Miller MW, Molloy PP, Mumby PJ, Nagelkerken I, Nemeth M, Navas-Camacho R, Pitt J, Polunin NVC, Reyes-Nivia MC, Robertson DR, Rodríguez-Ramírez A, Salas E, Smith SR, Spieler RE, Steele MA, Williams ID, Wormald CL, Watkinson AR, Côté IM (2009) Recent region-wide declines in Caribbean reef fish abundance. Curr Biol 19:590–595
10	Syms C, Jones GP (2000) Disturbance, habitat structure, and the dynamics of a coral-reef fish community. Ecology 81:2714–2729
11	Vergés A, Vanderklift MA, Doropoulos C, Hyndes GA (2011) Spatial patterns in herbivory on a coral reef are influenced by structural complexity but not by algal traits. PLoS One 6:e17115
12	Wilson SK, Graham NAJ, Pratchett MS, Jones GP, Polunin NVC (2006) Multiple disturbances and the global degradation of coral reefs: are reef fishes at risk or resilient? Glob Chang Biol 12:2220–2234

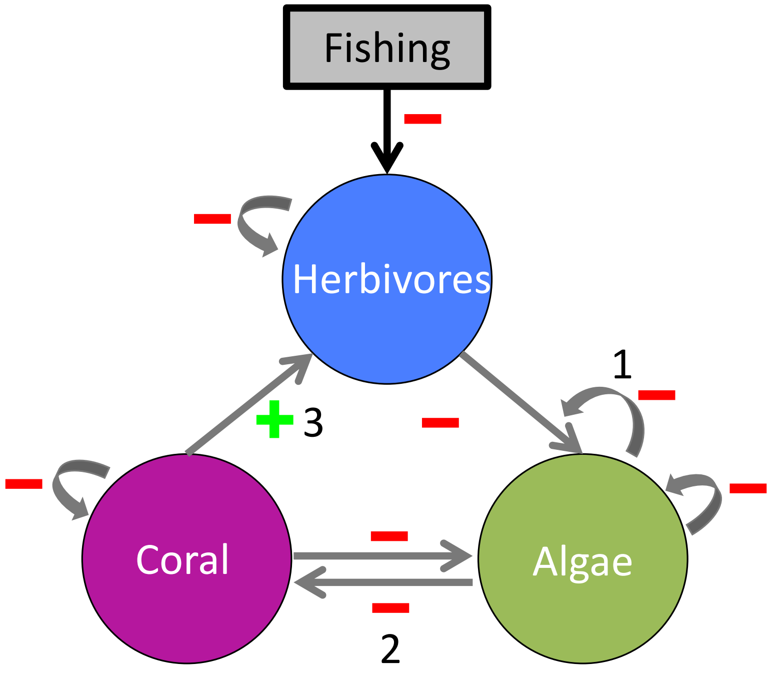

Figures

Figure A. Diagram of the model from van de Leemput et al. (2016), which we used as our baseline model to expand upon. The van de Leemput model has coral, herbivores, and algae as three state variables, and they each have density-dependent negative feedbacks to prevent unlimited population growth. This model also incorporated three de-stabilizing positive feedbacks: 1) decreased herbivory rate with increased algal cover via a Holling type II functional response for the herbivore, 2) negative effects of algal cover on coral recruitment and growth, and 3) increased herbivore carrying capacity with increased coral cover.

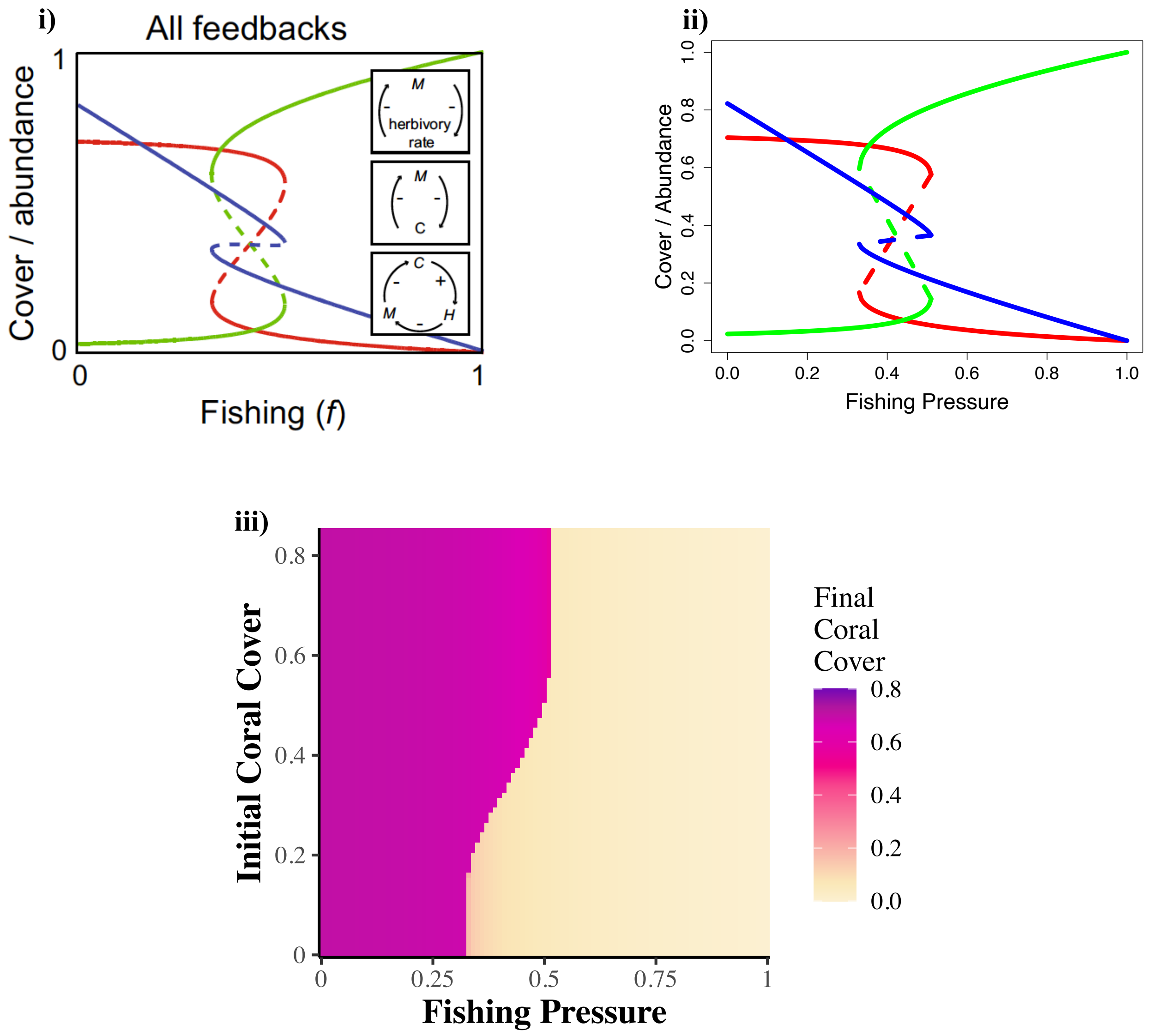

Figure B. Hysteresis plot from van de Leemput et al. (2016) (panel i), and our recreation of it ('basic model') using their equations, initial conditions, and parameter values coded in R (panel ii). Blue, red, and green lines represent herbivores, coral, and algae, respectively. iii) We also examined the bistability of our recreation of the van de Leemput et al. (2016) model ('basic model') for a range of fishing pressures and initial coral cover values at the start of a simulation. Each simulation of the model was run to steady state.

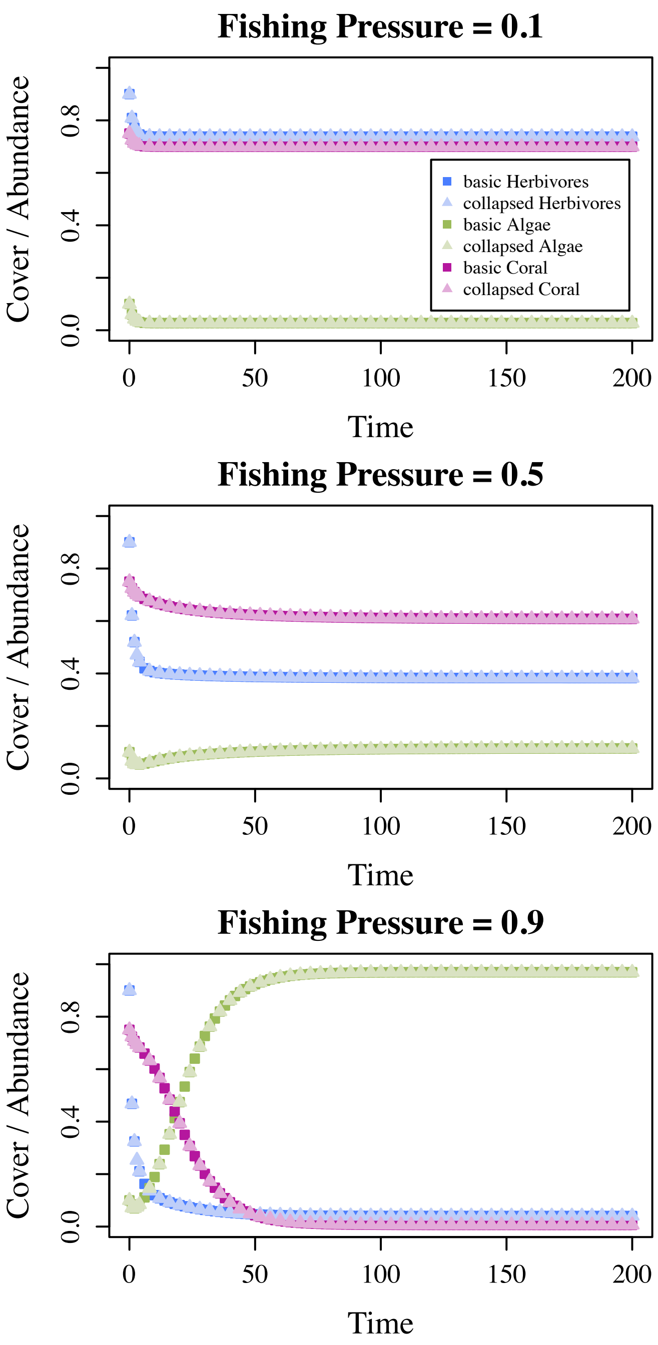

Figure C. Comparisons of 'basic model' and 'collapsed model'. We compared the outputs of our recreation of the van de Leemput et al. (2016) model ('basic model') (Table A) and our model using the "collapsed" initial conditions and parameter values from Table B. We compare outputs under 3 levels of fishing pressure: 0.1, 0.5, and 0.9.

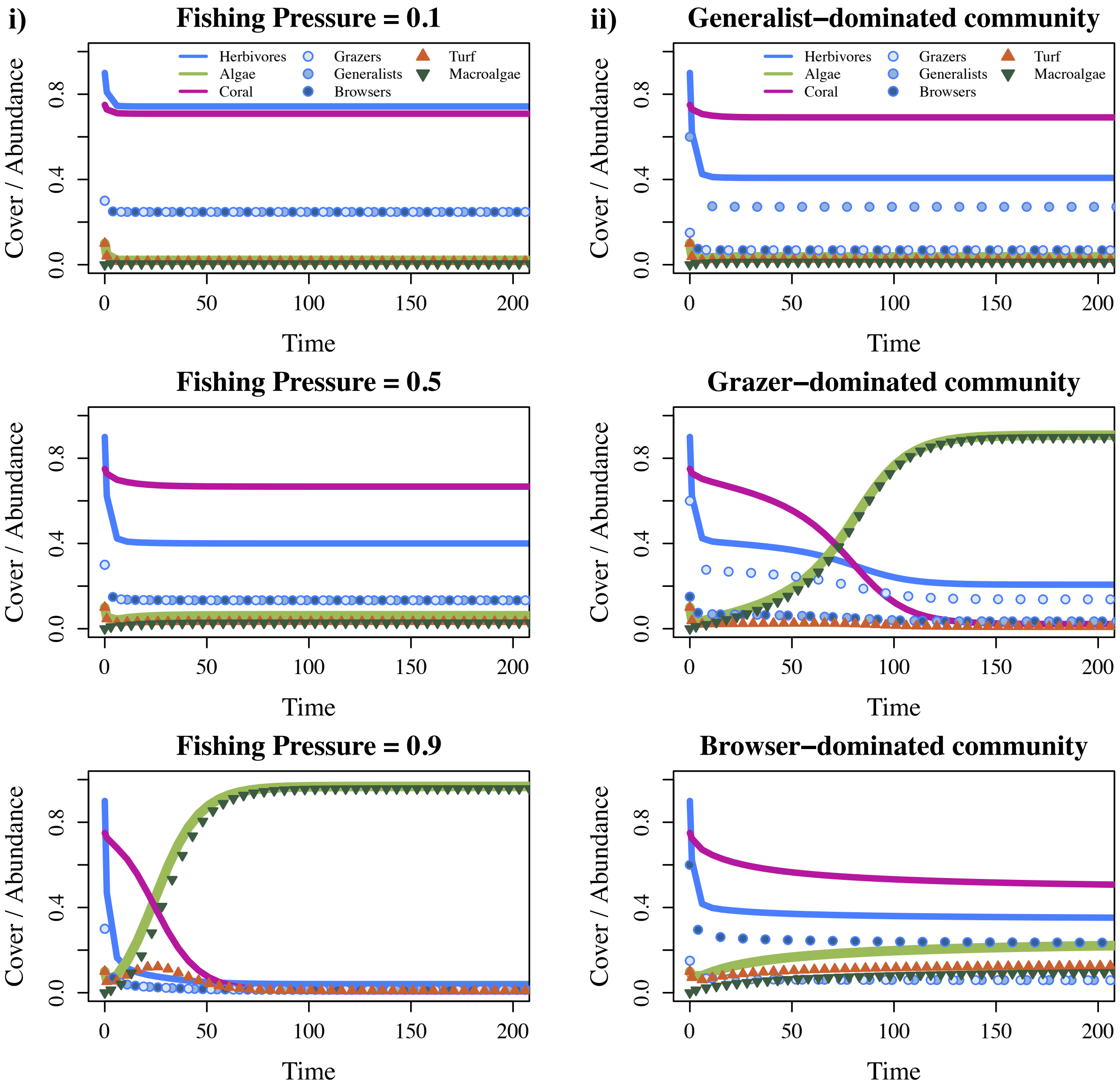

Figure D. Model output over time for varying fishing pressure and herbivore community scenarios. i) Output of our full model under 3 levels of fishing pressure (0.1, 0.5, 0.9) for an herbivore community evenly distributed between grazers, browsers, and generalists, and initial conditions and parameter values given in Table S2. ii) Output of our model under 3 herbivore community composition scenarios (generalist-dominated, grazer-dominated, browser-dominated), for fishing pressure = 0.5. For both panels A and B, the Herbivores and Algal state variables are sums of their corresponding state variables (i.e., H = G+B+R, and A = T+M).

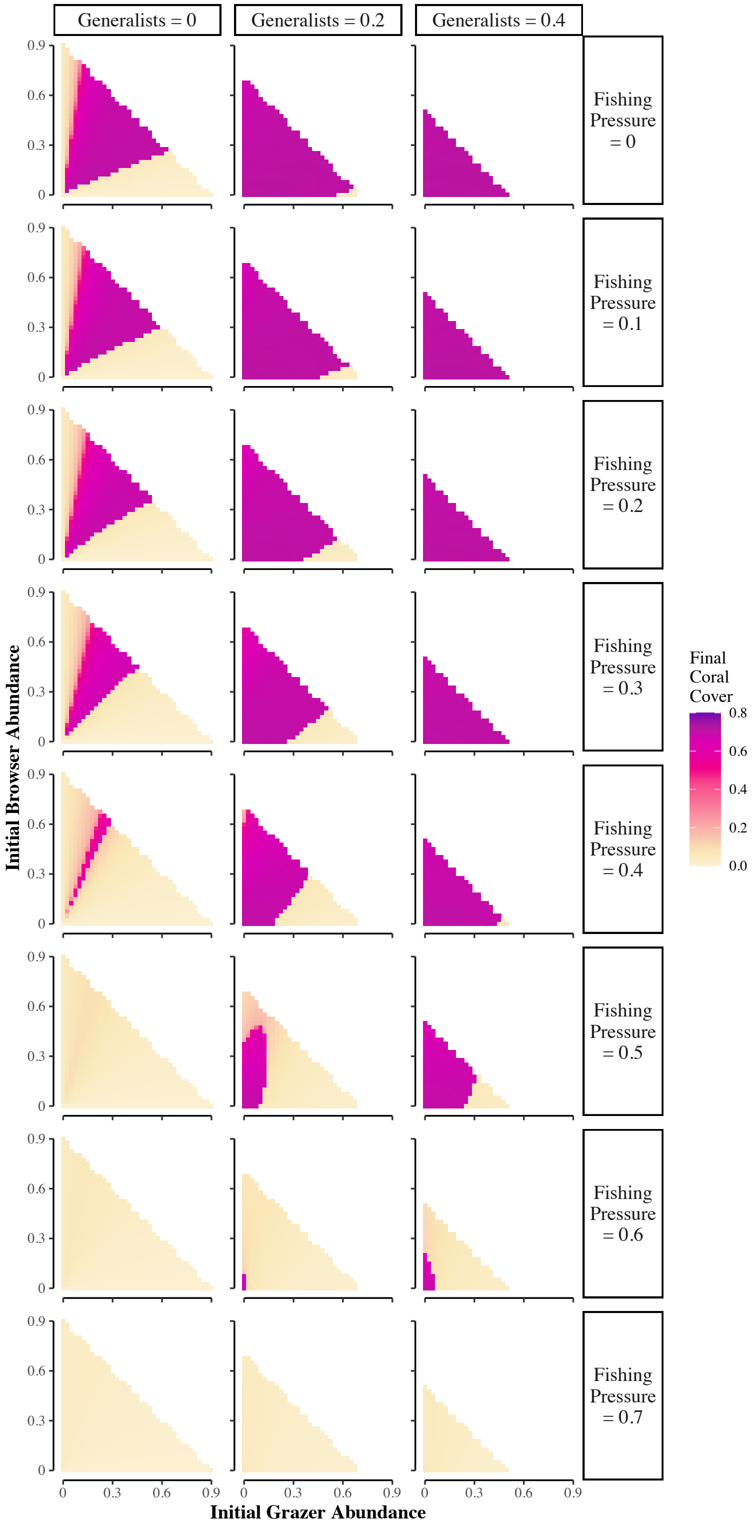

Figure E. Q1 final coral cover full heatmap results. Heatmaps showing how the initial abundances of grazer and browser herbivorous fish affect the steady state coral cover for fishing pressures ranging from 0 to 0.7 (rows) and for initial generalist abundances set to 0, 0.2, or 0.4 (columns). Other initial conditions include: $C_{t=0} = 0.15$, $T_{t=0} = 0.7$, and $M_{t=0} = 0$.

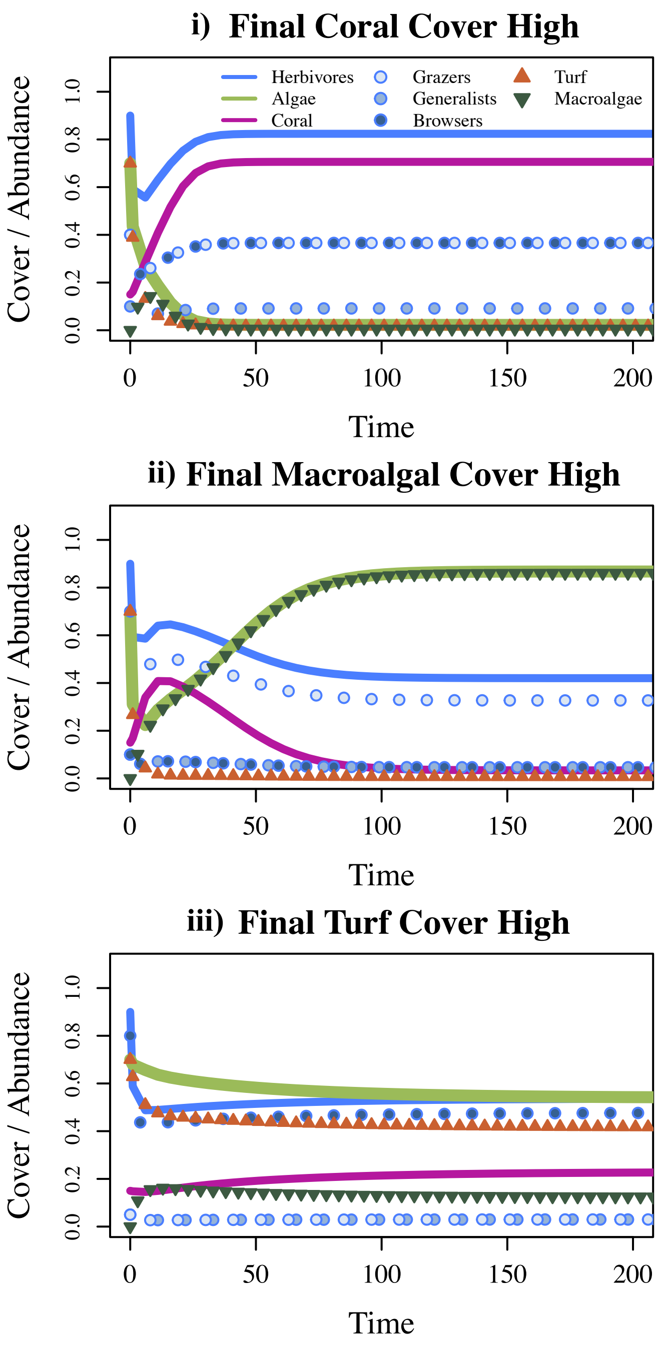

Figure F. Output of our model over time for scenarios examined in Question 1. Specifically, we show output from scenarios where the benthic group that ends up with the highest final cover is either i) coral, ii) macroalgae, or iii) turf. For all panels $C_{t=0} = 0.15$, $T_{t=0} = 0.7$, $M_{t=0} = 0$, and fishing pressure = 0. For i) Initial fish community balanced between browsers and grazers: $G_{t=0} = B_{t=0} = 0.4$, and $R_{t=0} = 0.1$; for ii) Initial fish community skewed toward grazers $G_{t=0} = 0.7$, $B_{t=0} = 0.1$, and $R_{t=0} = 0.1$; and for iii) Initial fish community skewed toward browsers: $G_{t=0} = 0.05$ and $B_{t=0} = 0.8$, and $R_{t=0} = 0.05$. The Herbivores and Algal state variables are sums of their corresponding state variables (i.e., H = G+B+R, and A = T+M).

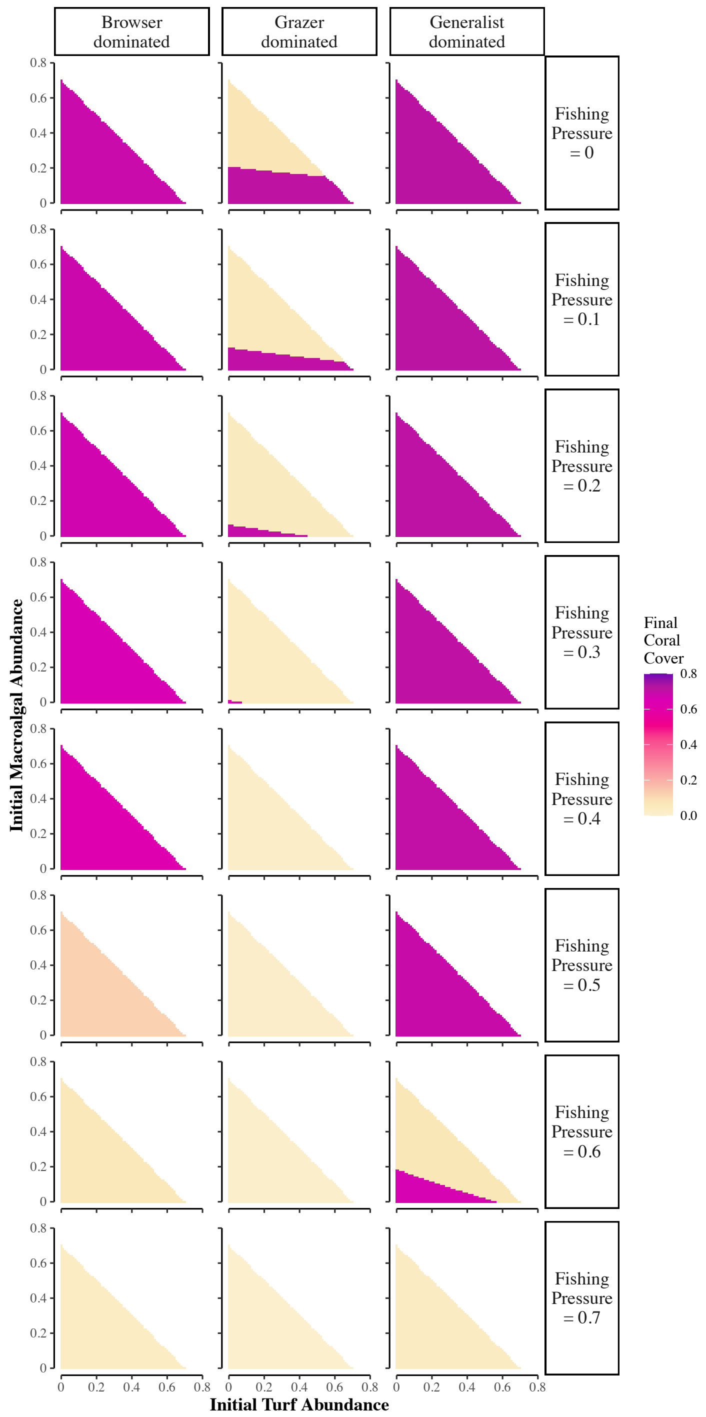

Figure G. Q2 final coral cover full heatmap results. Heatmaps showing how the initial cover of turf and macroalgae affects steady state coral cover for fishing pressures from 0 to 0.7 (rows) and for initial herbivore abundances set to 1 of 3 scenarios (columns): 1) **browser-dominated**: browsers = 0.6, generalists = 0.15, grazers = 0.15; 2) **grazer-dominated**: grazers = 0.6, generalists = 0.15, browsers = 0.15; or 3) **generalist-dominated**: generalists = 0.6, grazers = 0.15, browsers = 0.15. Total initial benthic cover is restricted to ≤0.85, and initial coral cover ($C_{t=0}$) = 0.15; therefore, the amount of unoccupied space changes for each combination of initial turf and macroalgae abundance.

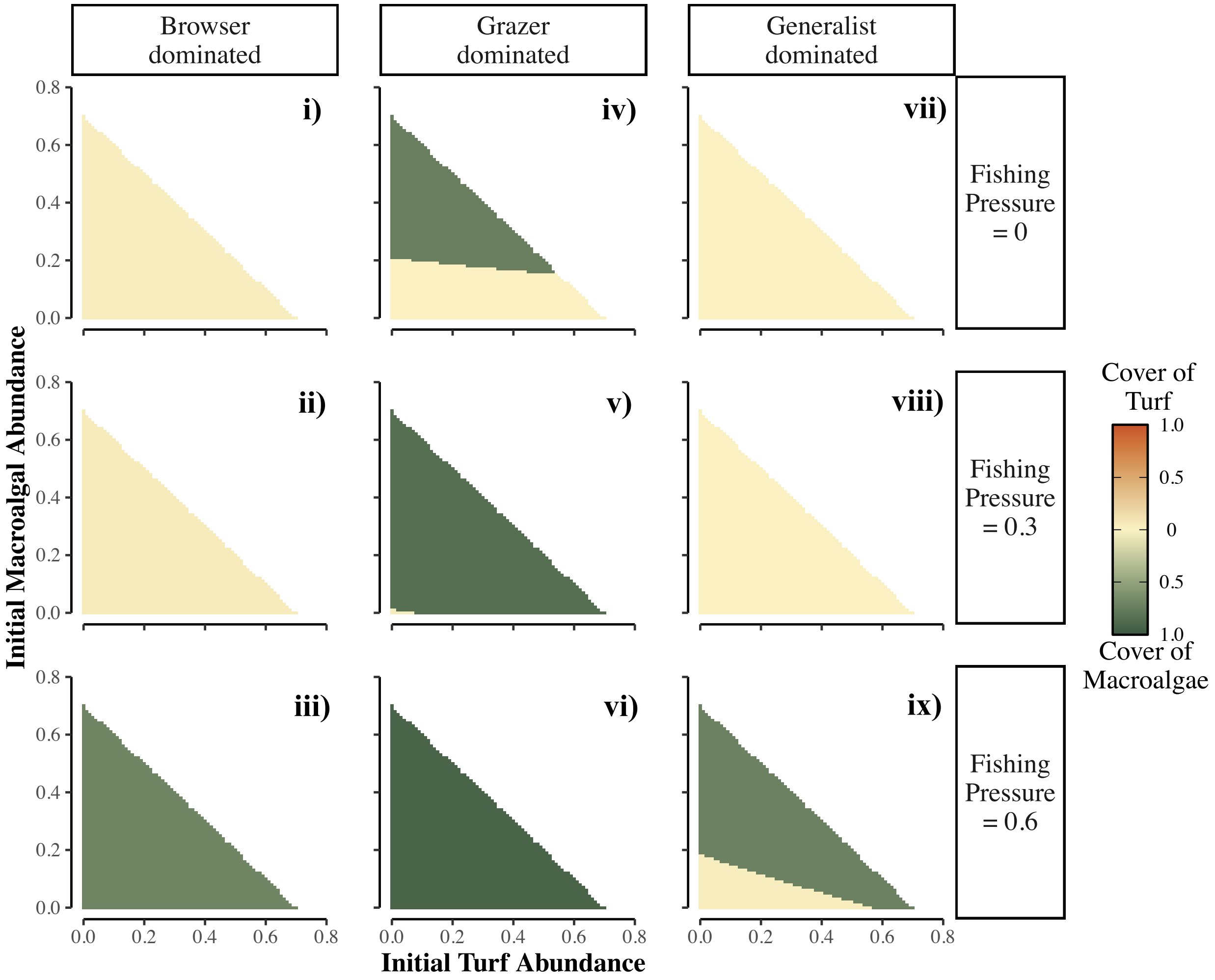

Figure H. Q2 final macroalgal and turf cover full heatmap results. Heatmaps showing final cover of turf or macroalgae for the same scenarios as Fig. 5. Specifically, these panels examine the initial cover of turf and macroalgae for fishing pressures ranging from 0 to 0.6 (rows) and for initial herbivore proportions set to 1 of 3 scenarios: 1) browser-dominated (A-C) ; 2) grazer-dominated (D-F); or 3) generalist-dominated (G-I). For each scenario, the heatmap indicates the final cover of turf or macroalgae, depending on which had the higher final cover. If the final cover of turf was higher, it is shown as an orange color. If the final cover of macroalgae was higher, it is shown as a green color.

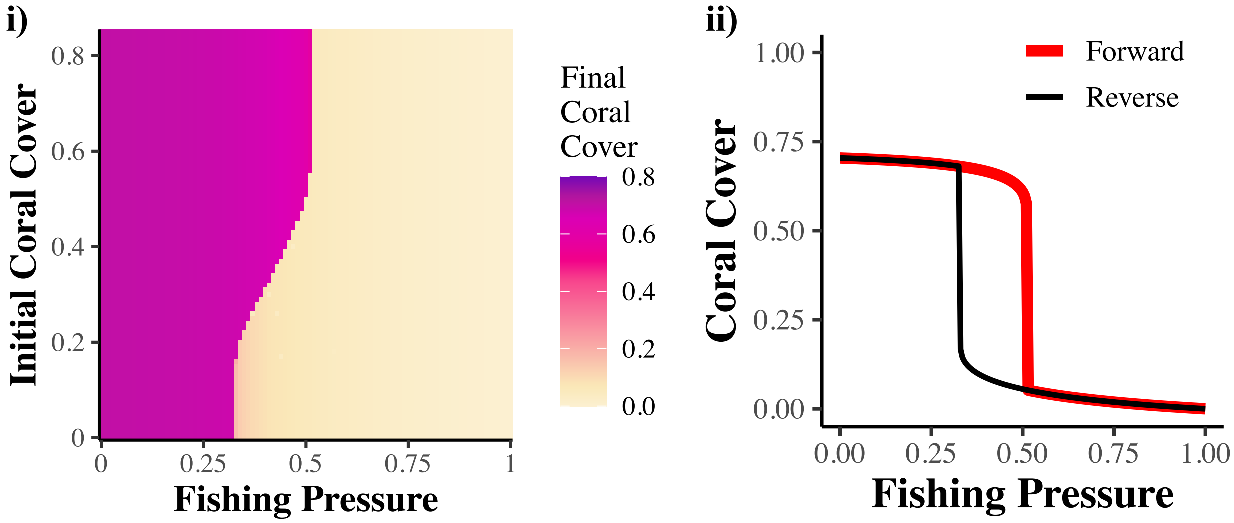

Figure I. Bistability and hysteresis plots for our 'collapsed' model. Bistability and hysteresis plots for our model using the parameter values and initial conditions for which it collapses to the van de Leemput et al (2016) model. Bistability plot (i) showing final coral cover for each combination of fishing pressure and initial coral cover conditions. Bistability is evident when, for a given fishing pressure, both low (<0.2) and high (>0.6) final coral covers occur, depending only upon the initial coral cover. Hysteresis plot (ii) showing the final coral cover as fishing pressure is increased (forward) or decreased (reverse). Hysteresis is evident when there is a range of fishing pressures where the forward (red) and reverse (black) lines do not overlap.

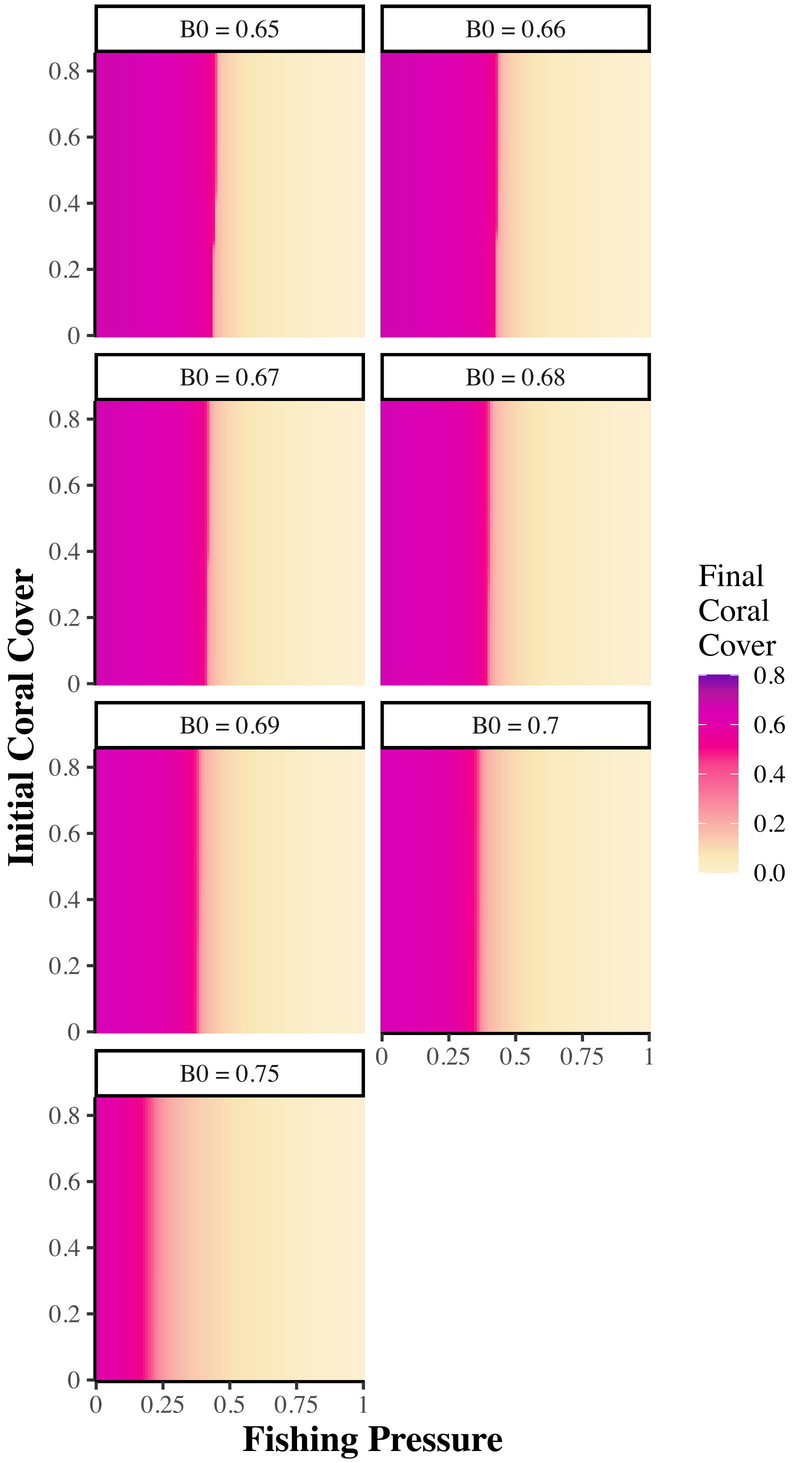

Figure J. Bistability plots for different B0 (initial abundances of browsers). Bistability plots showing final coral cover for combinations of fishing pressure and initial coral cover conditions. Different panels show B0, which is the different initial abundances of the browser herbivore functional group (B0 = 0.65, 0.66, 0.67, 0.68, 0.69, 0.70, and 0.75). Bistability no longer occurs when B0 >= 0.70.

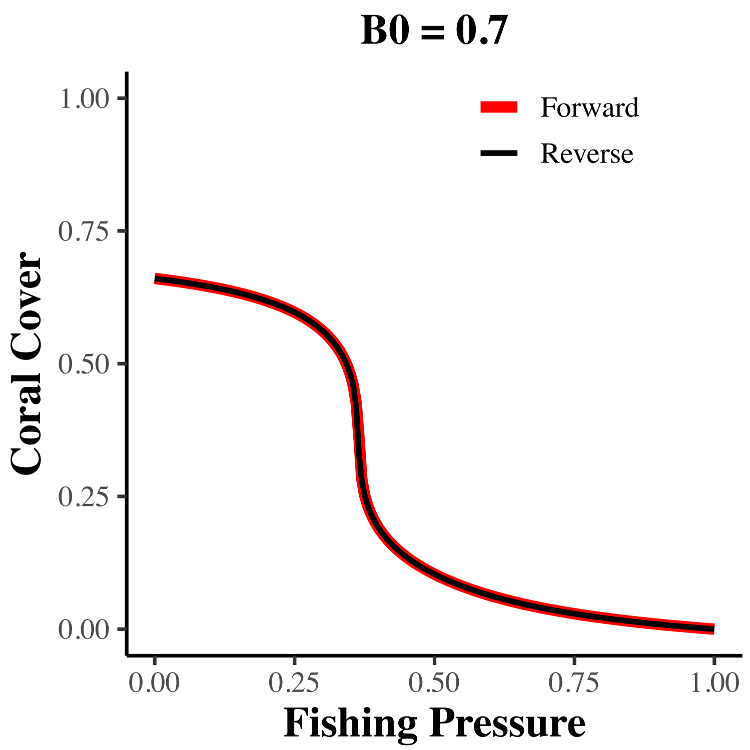

Figure K. Hysteresis plot for B0 = 0.7. Hysteresis plot showing the final coral cover as fishing pressure is increased (forward) or decreased (reverse). Hysteresis is evident when there is a range of fishing pressures where the forward (red) and reverse (black) lines do not overlap. In this scenario, with an initial browser abundance of 0.7, there is no longer hysteresis in this coral reef system.

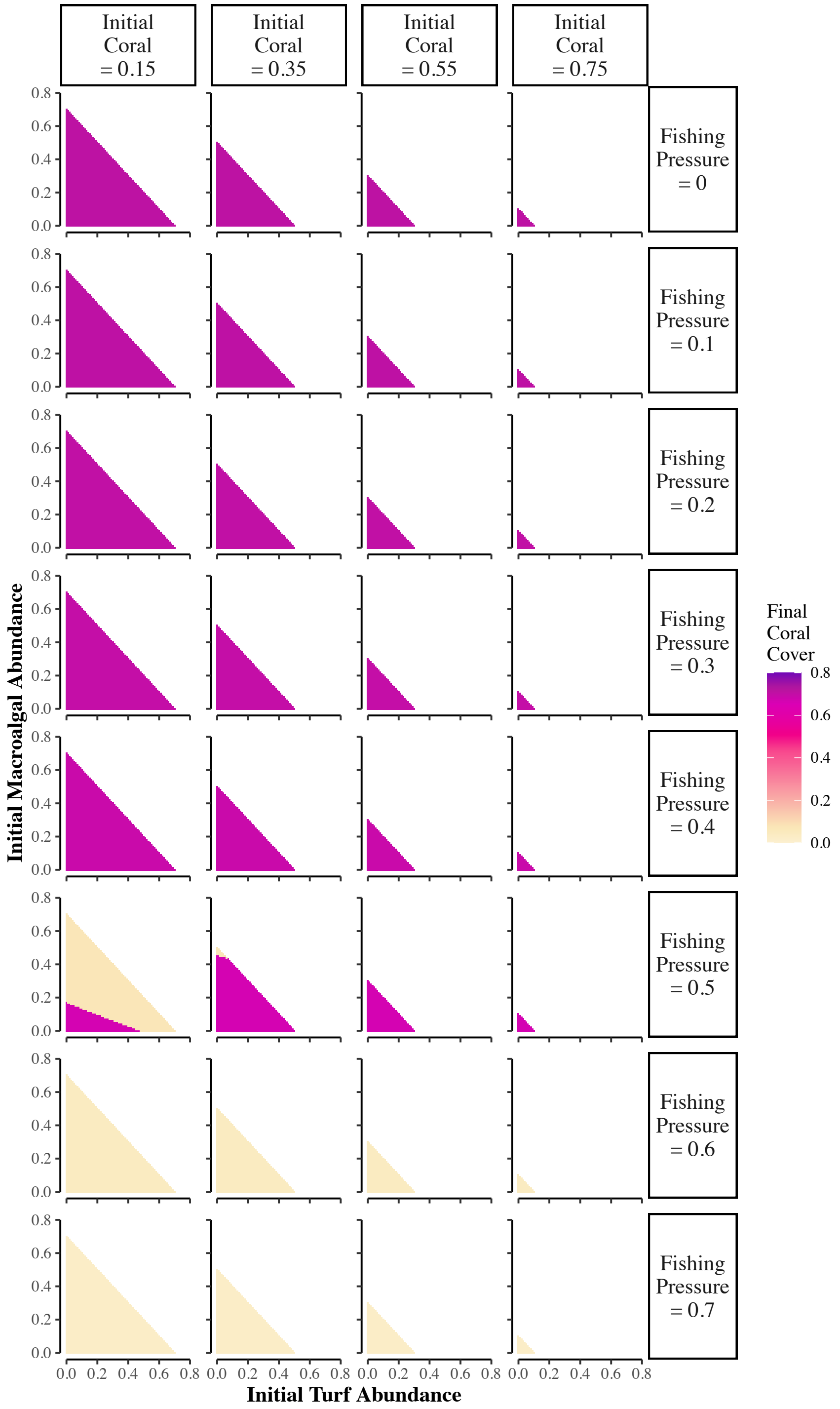

Figure L. Supplemental Question 1 final coral cover heatmap results. Heatmaps showing how initial cover of turf and macroalgae affect steady state coral cover for fishing pressures ranging from 0 to 0.7 (rows) and for initial coral cover set to 0.15, 0.35, 0.55, and 0.75 (columns). Total initial benthic cover is restricted to ≤0.85; so, as initial coral cover increases, the initial cover of turf+macroalgae has to decrease accordingly, resulting in smaller response spaces in the panels. Initial herbivore abundances each set to 0.3.

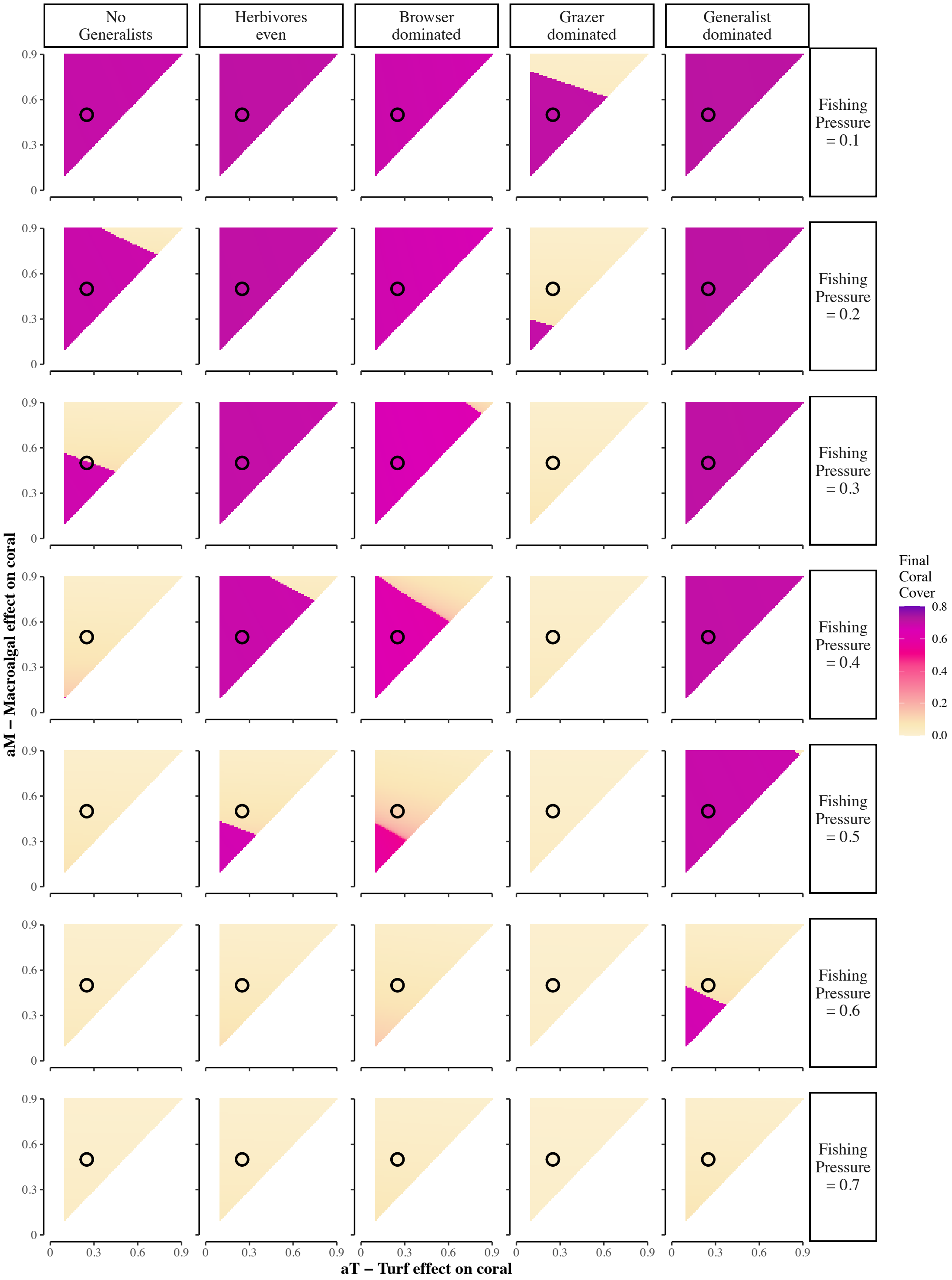

Figure M. Sensitivity analysis for competitive effects of macroalgae and turf on coral (parameters α_M and α_T). α_M and α_T values ranging from 0.1 – 0.9 and under varying fishing pressure conditions (0.1 – 0.7) and herbivore community scenarios (no generalists, even herbivore community, browser-dominated, grazer-dominated, and generalist-dominated). The circle in each panel indicates the parameter values we used in our model analyses.

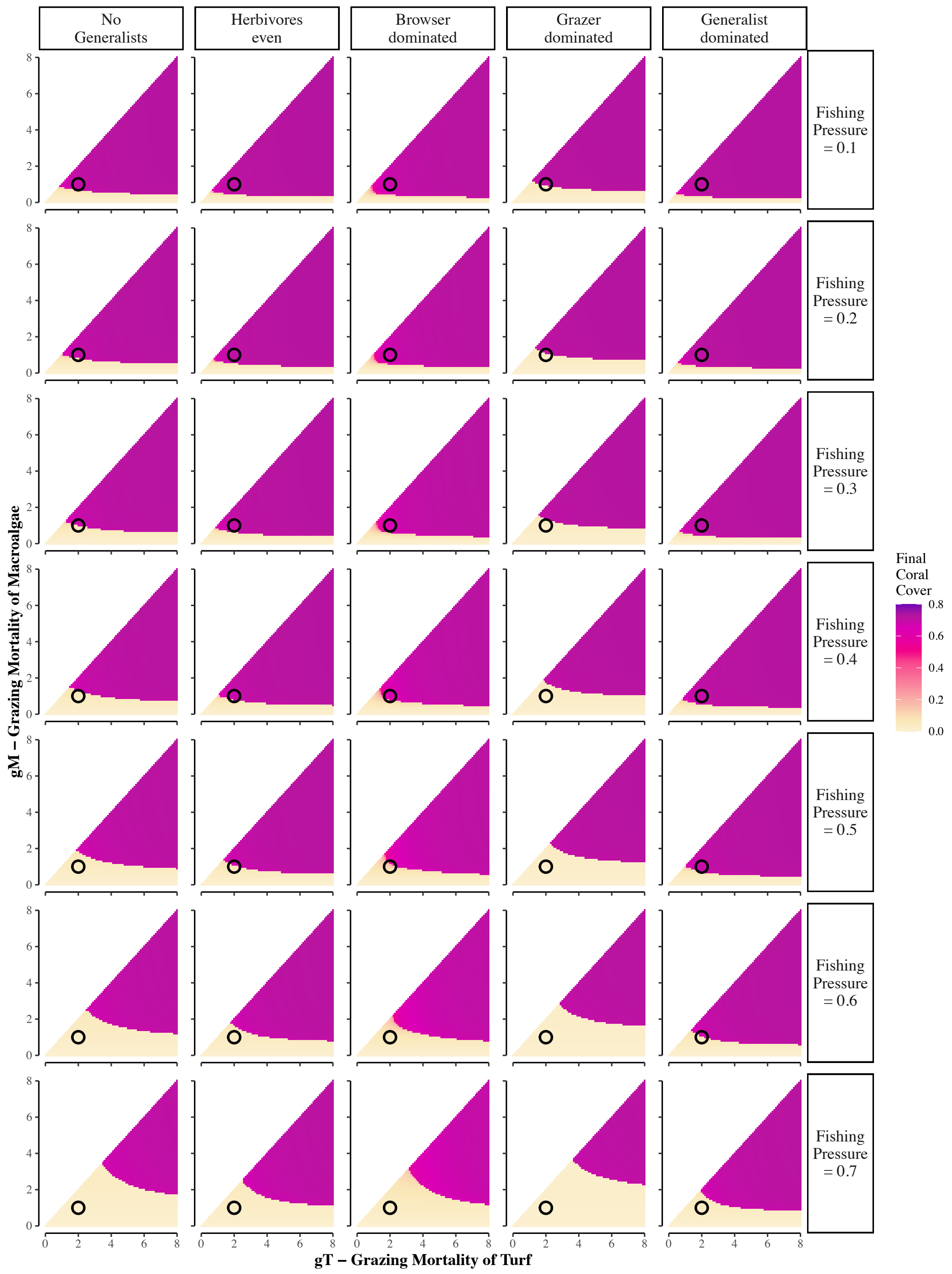

Figure N. Sensitivity analysis for mortality of macroalgae and turf from herbivory (parameters g_M and g_T). g_M and g_T values ranging from 0 – 8 and under varying fishing pressures (0.1 – 0.7) and herbivore community scenarios (no generalists, even herbivore community, browser-dominated, grazer-dominated, and generalist-dominated). The circle in each panel indicates the parameter values we used in our model analyses.

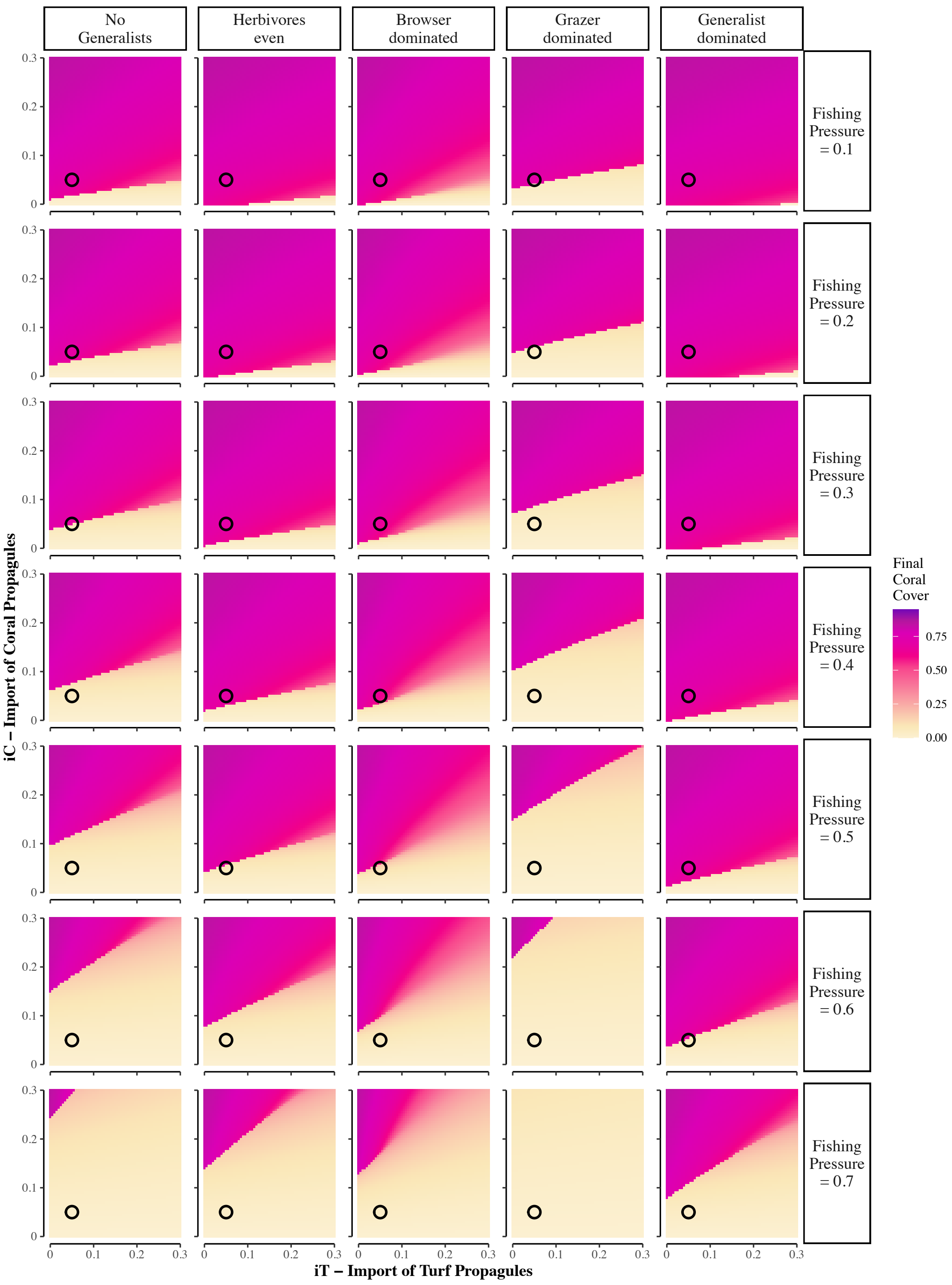

Figure O. Sensitivity analysis for import of coral and turf propagules (parameters i_C and i_T). i_C and i_T values ranging from 0 – 0.3 and under varying fishing pressure conditions (0.1 – 0.7) and herbivore community scenarios (no generalists, even herbivore community, browser-dominated, grazer-dominated, and generalist-dominated). The circle in each panel indicates the parameter values we used in our model analyses.

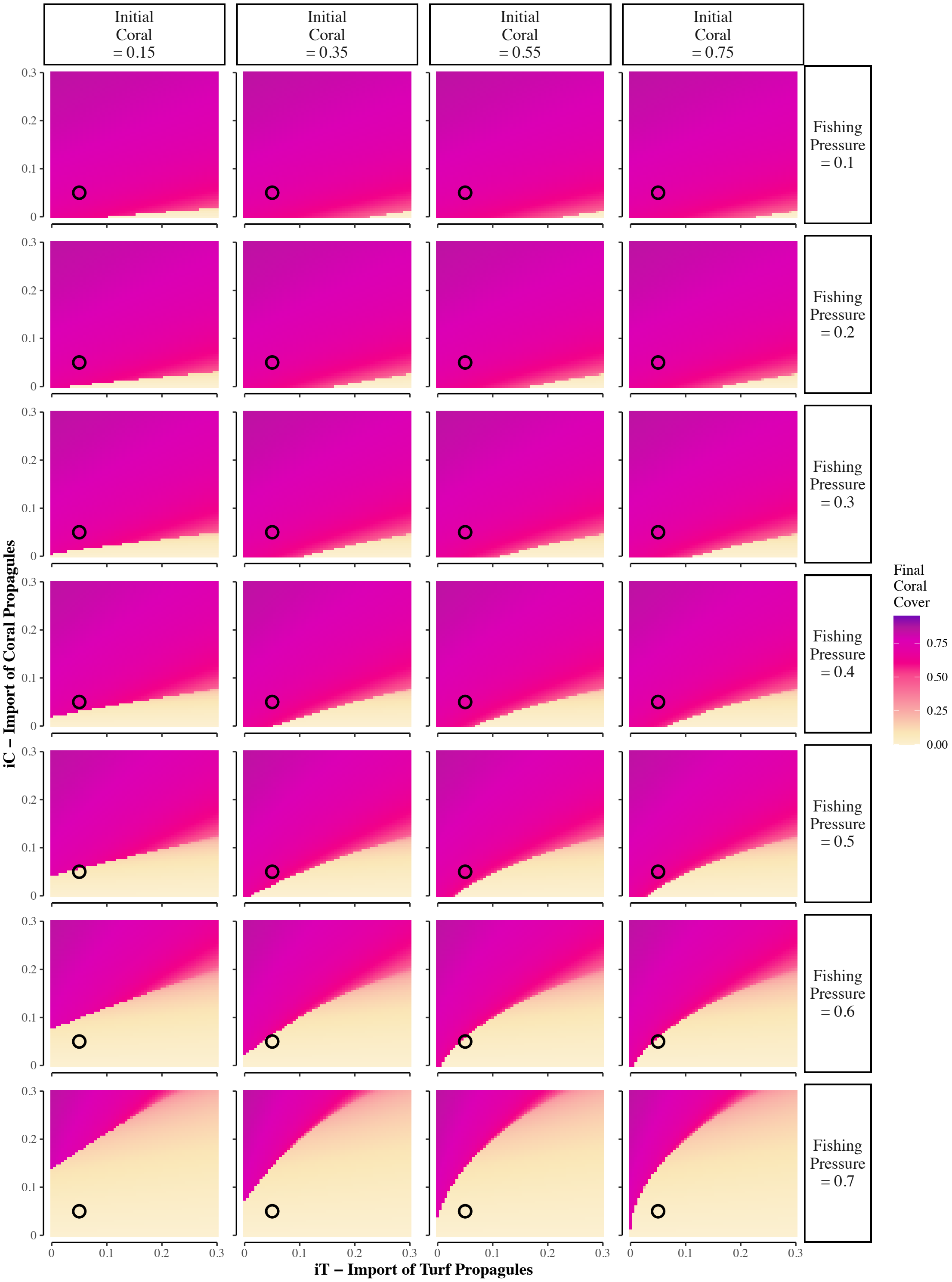

Figure P. Sensitivity analysis for import of coral and turf propagules (parameters i_C and i_T). i_C and i_T values ranging from 0 – 0.3 when starting with different initial coral cover conditions (initial coral = 0.75, 0.55, 0.35, and 0.15) under varying fishing pressures (0.1 – 0.7). The circle in each panel indicates the parameter values we used in our model analyses.

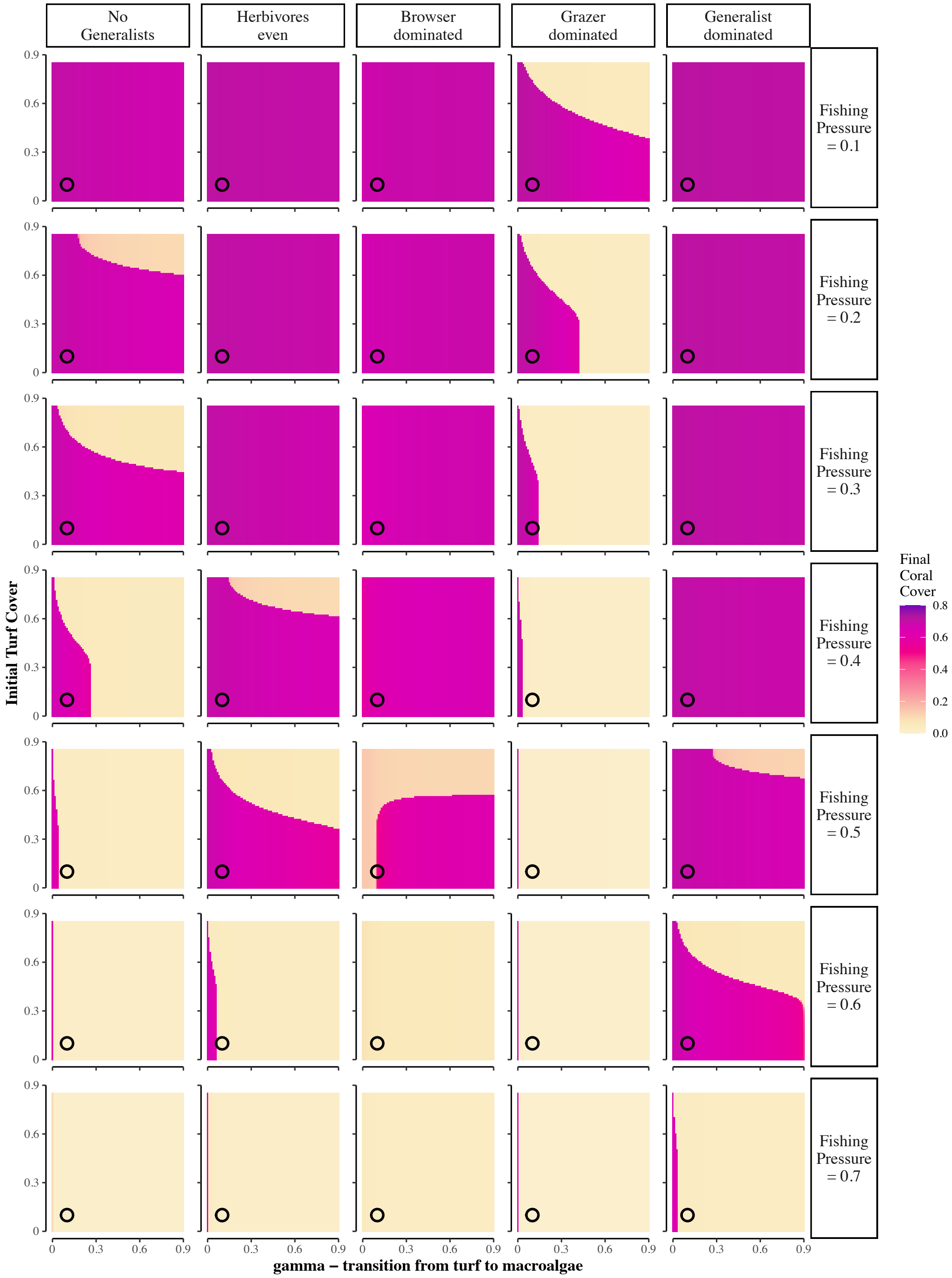

Figure Q. Sensitivity analysis for transition from turf to macroalgae (parameter gamma γ). Gamma (γ) values ranging from 0 – 0.9, and we explored these values in relation to initial turf cover (T0 values from 0 – 0.85), and under varying fishing pressures (0.1 – 0.7) and herbivore community scenarios (no generalists, even herbivore community, browser-dominated, grazer-dominated, and generalist-dominated). The circle in each panel indicates the parameter values we used in our model analyses.

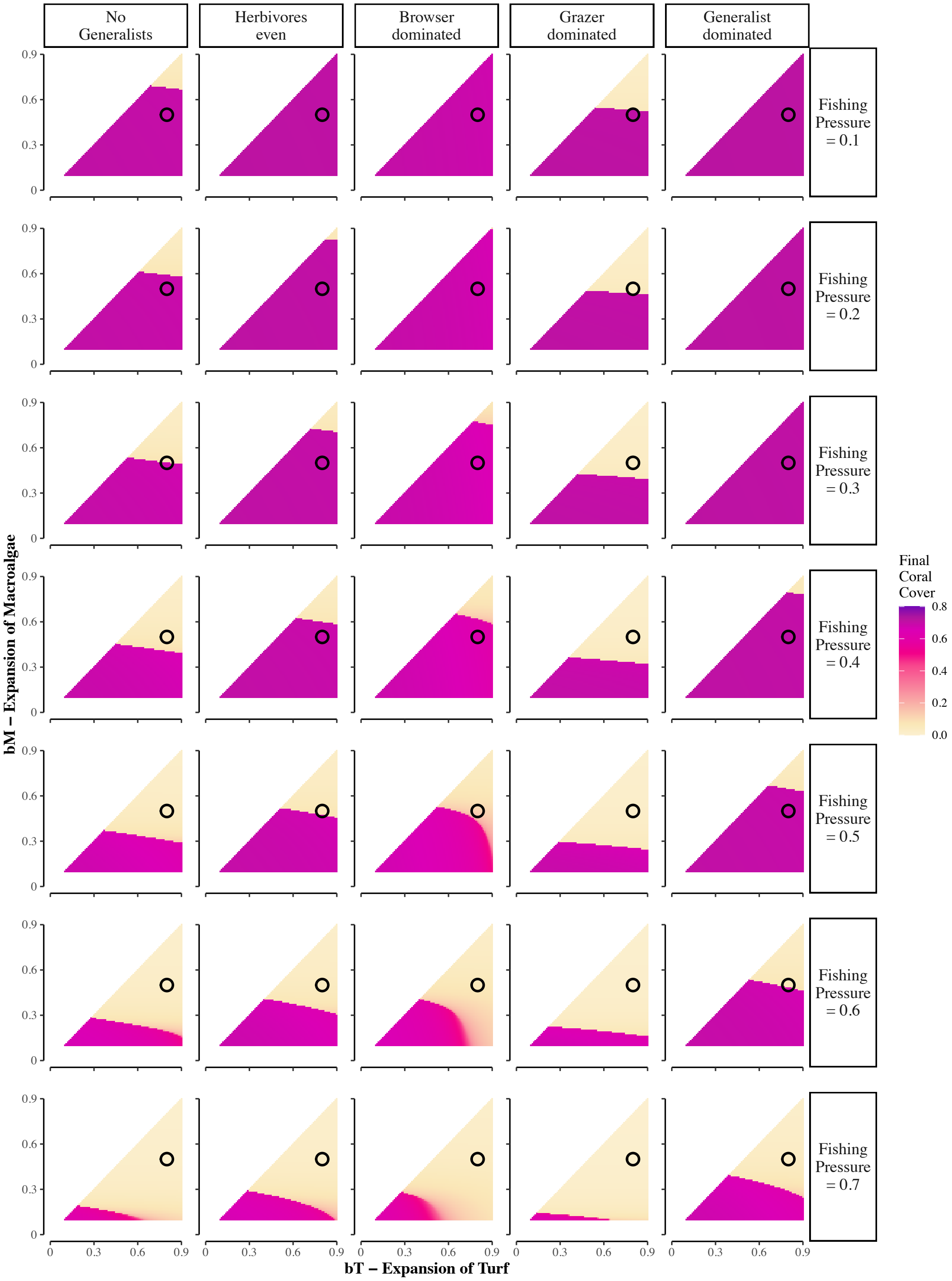

Figure R. Sensitivity analysis for expansion of turf and macroalgae (parameters b_T and b_M). Parameter b_T and b_M values ranging from 0.1 – 0.9 under varying fishing pressures (0.1 – 0.7) and herbivore community scenarios (no generalists, even herbivore community, browser-dominated, grazer-dominated, and generalist-dominated). The circle in each panel indicates the parameter values we used in our model analyses.

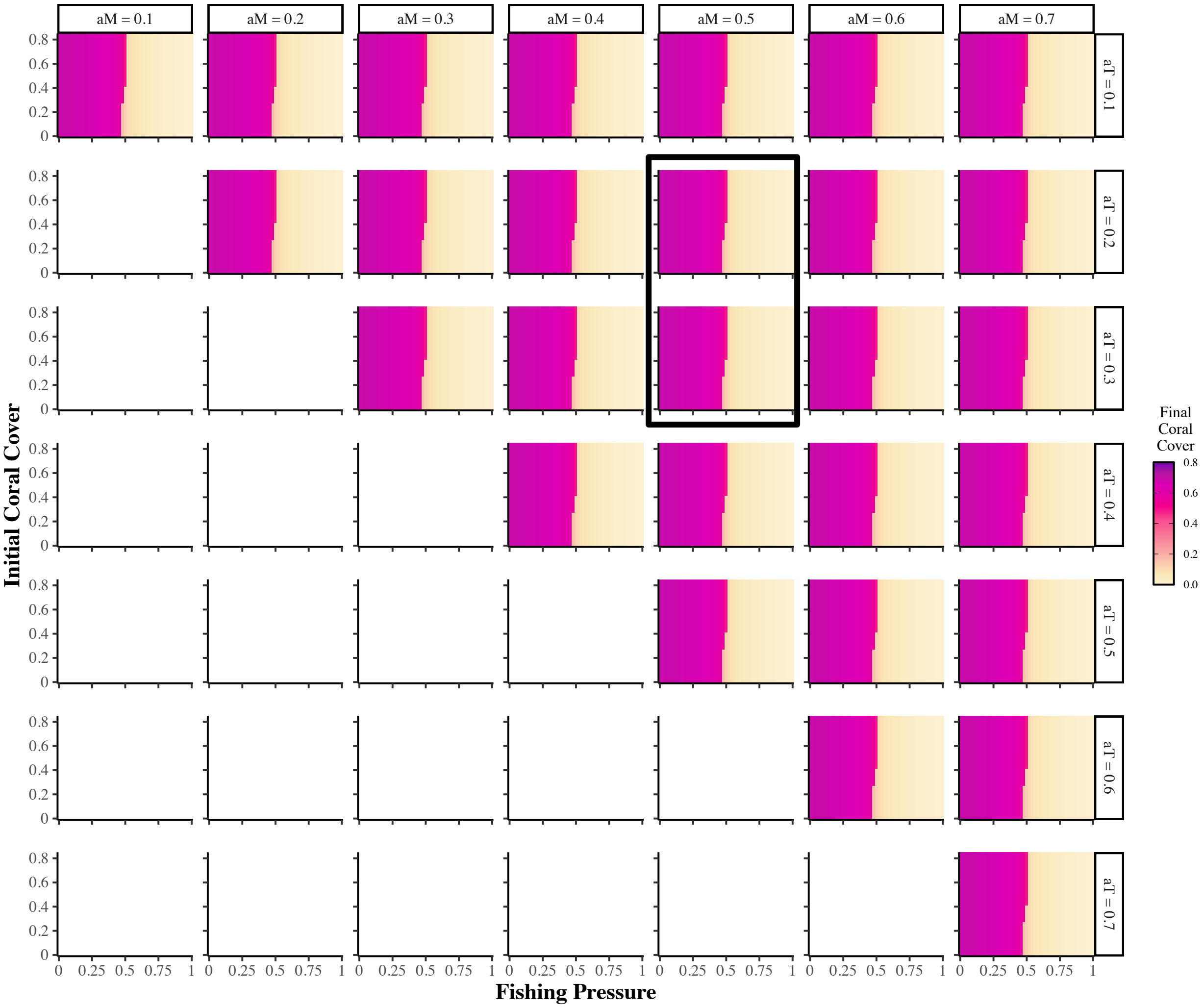

Figure S. Bistability sensitivity analysis for competitive effects of macroalgae and turf on coral (parameters α_M and α_T) in browser-dominated reefs. We examined α_M and α_T values ranging from 0.1 – 0.7 in increments of 0.1. The outlined panel indicates the parameter values we used in our model analyses ($\alpha_T = 0.25$ and $\alpha_M = 0.5$).

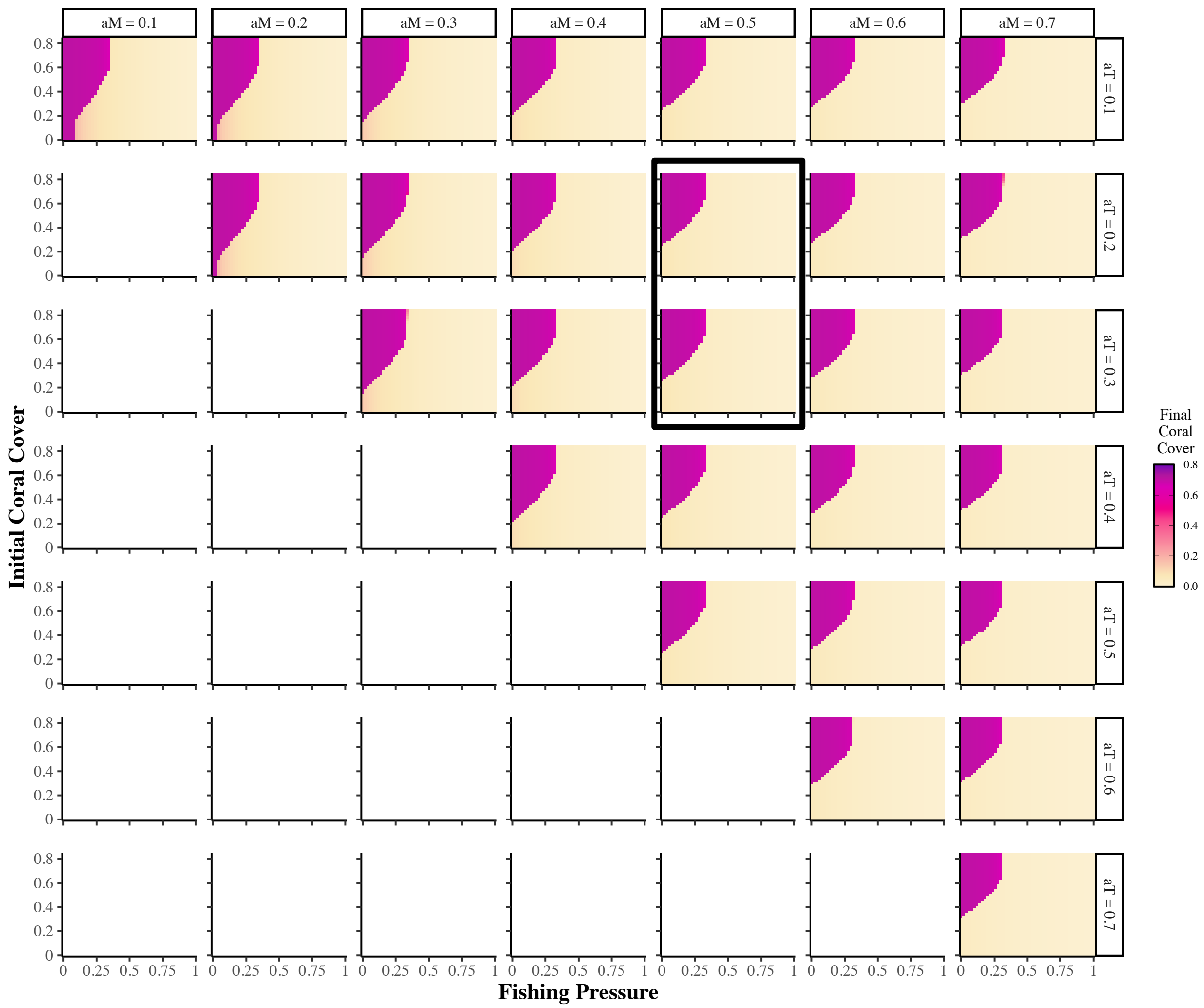

Figure T. Bistability sensitivity analysis for competitive effects of macroalgae and turf on coral (parameters α_M and α_T) for grazer-dominated reefs. We examined α_M and α_T values ranging from 0.1 – 0.7 in increments of 0.1. The outlined panel indicates the parameter values we used in our model analyses ($\alpha_T = 0.25$ and $\alpha_M = 0.5$).

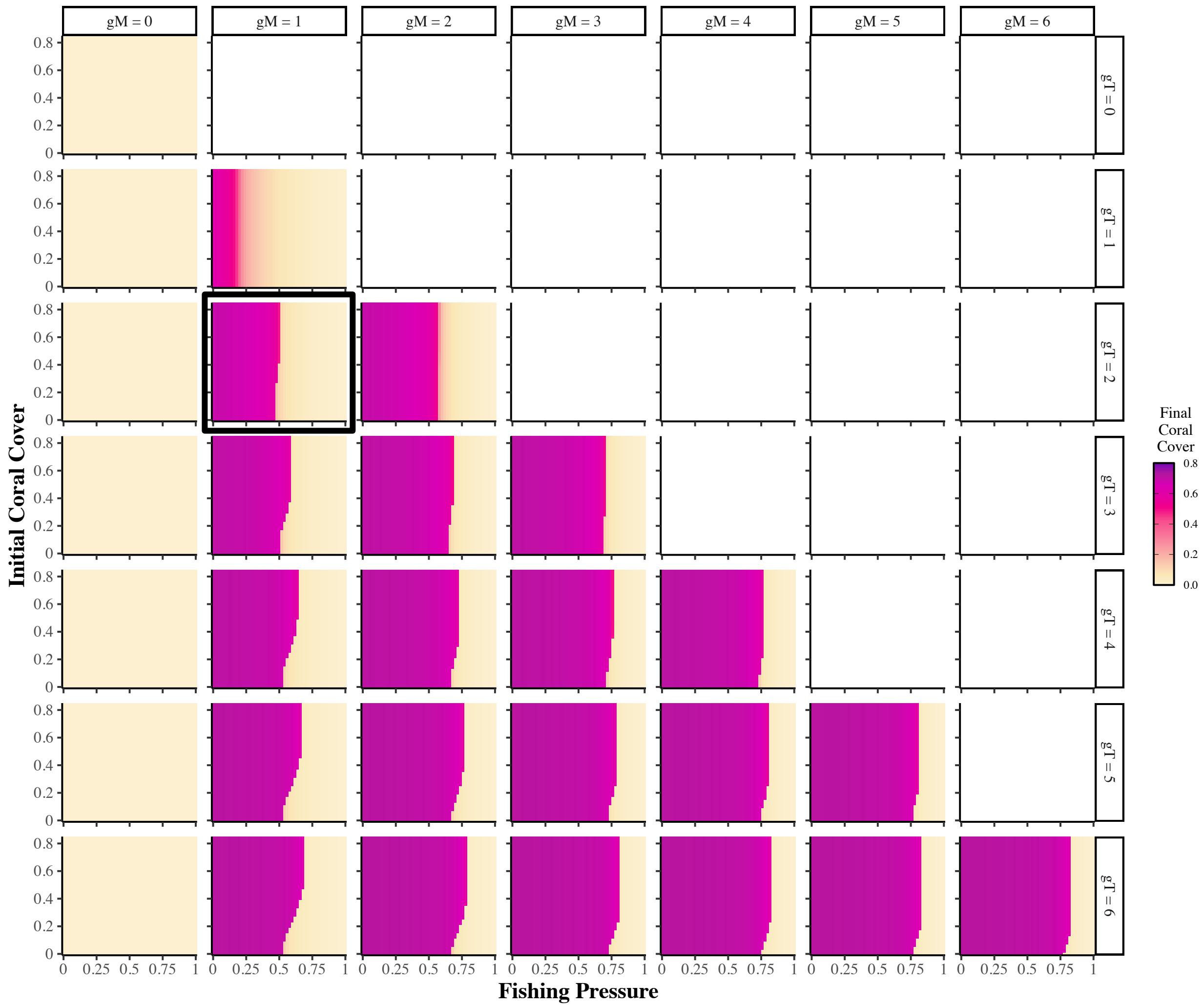

Figure U. Bistability sensitivity analysis for mortality of macroalgae and turf from herbivory (parameters g_M and g_T) for browser-dominated reefs. We examined g_M and g_T values ranging from 0 – 6 in increments of 1. The outlined panel indicates the parameter values we used in our model analyses (g_T = 2 and g_M = 1).

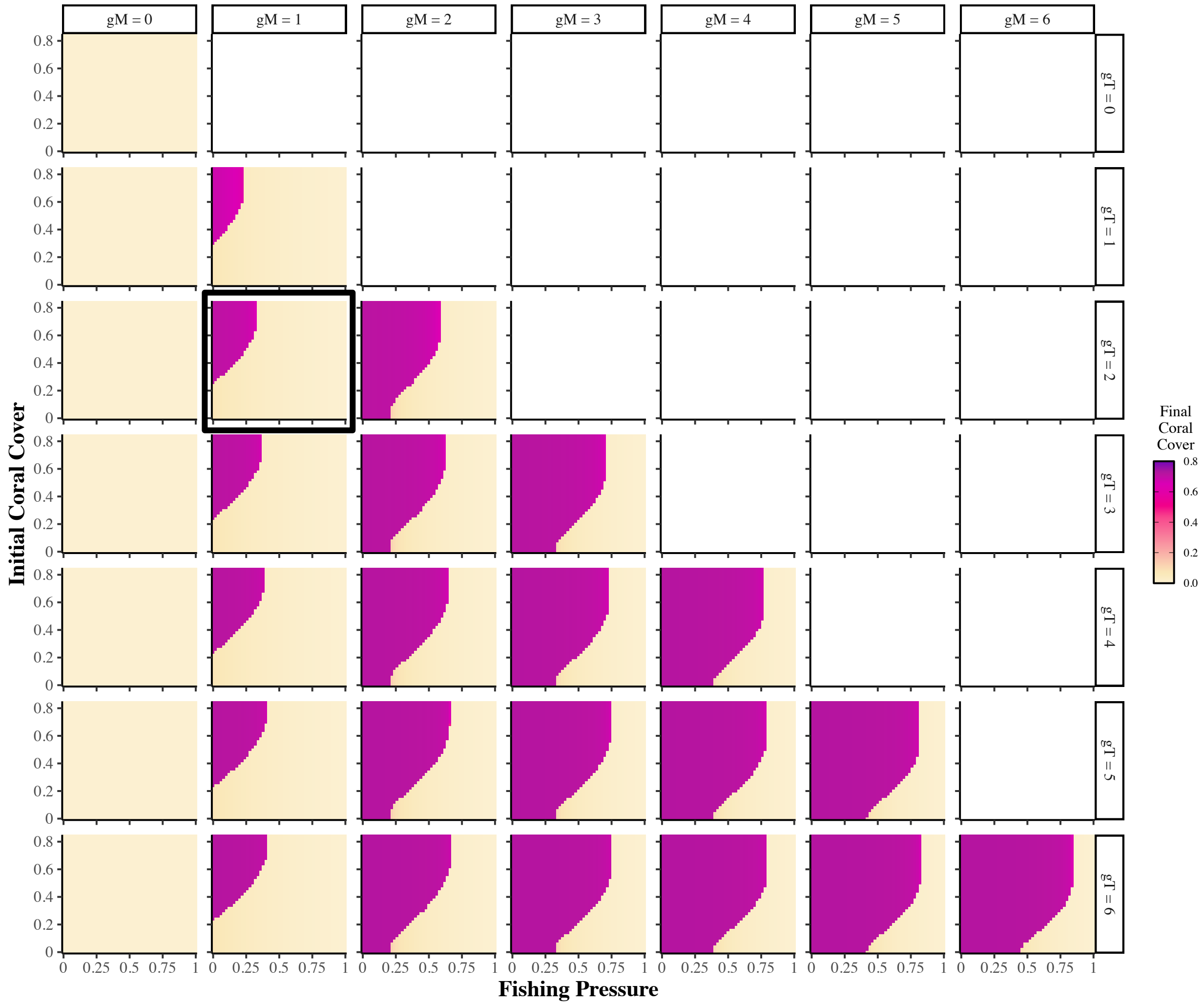

Figure V. Bistability sensitivity analysis for mortality of macroalgae and turf from herbivory (parameters g_M and g_T) for grazer-dominated reefs. We examined g_M and g_T values ranging from 0 – 6 in increments of 1. The outlined panel indicates the parameter values we used in our model analyses ($g_T = 2$ and $g_M = 1$).

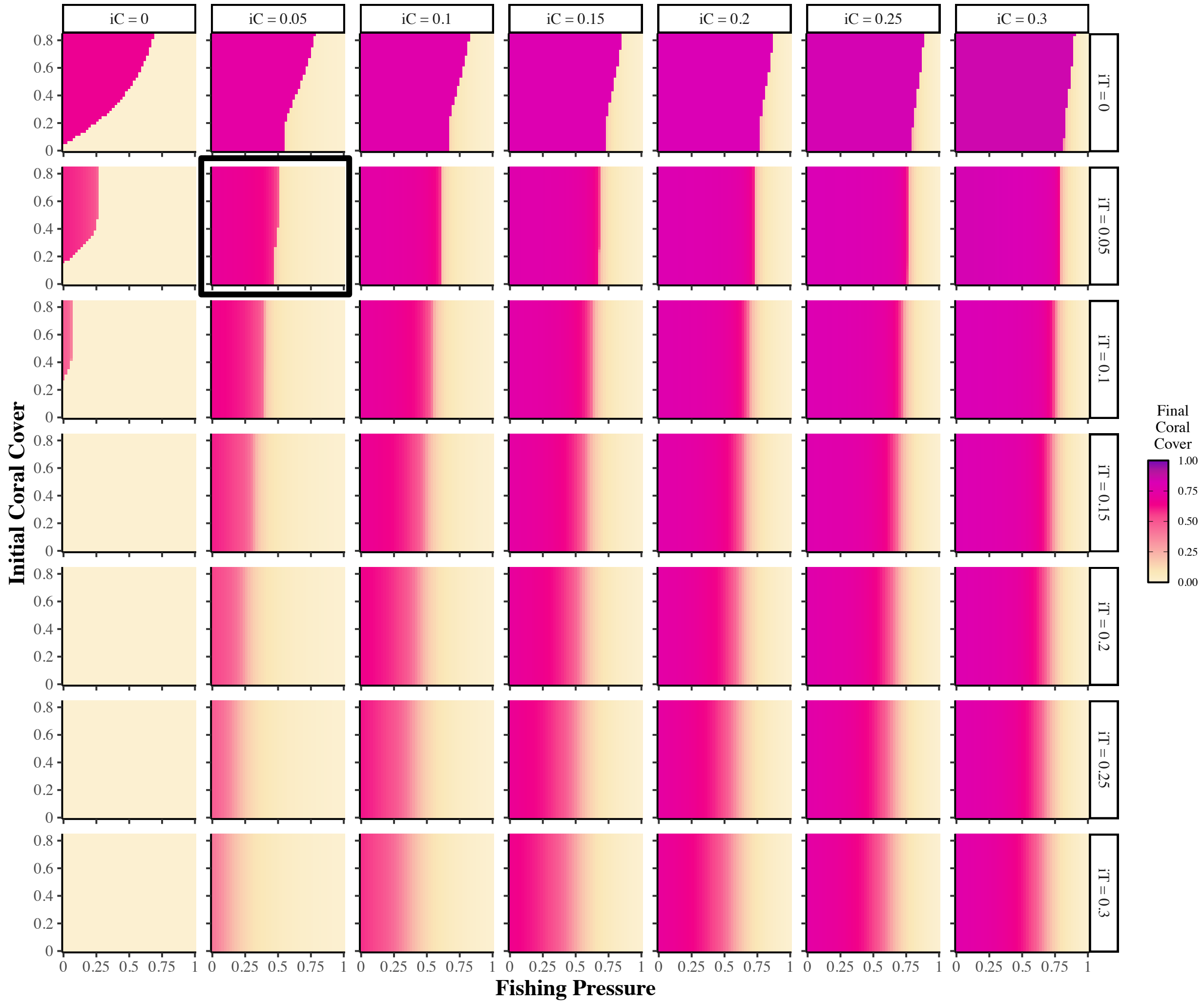

Figure W. Bistability sensitivity analysis for import of coral and turf propagules (parameters i_C and i_T) for browser-dominated reefs. We examined i_C and i_T values ranging from 0 – 0.3 in increments of 0.05. The outlined panel indicates the parameter values we used in our model analyses (i_C = 0.05 and i_T = 0.05).

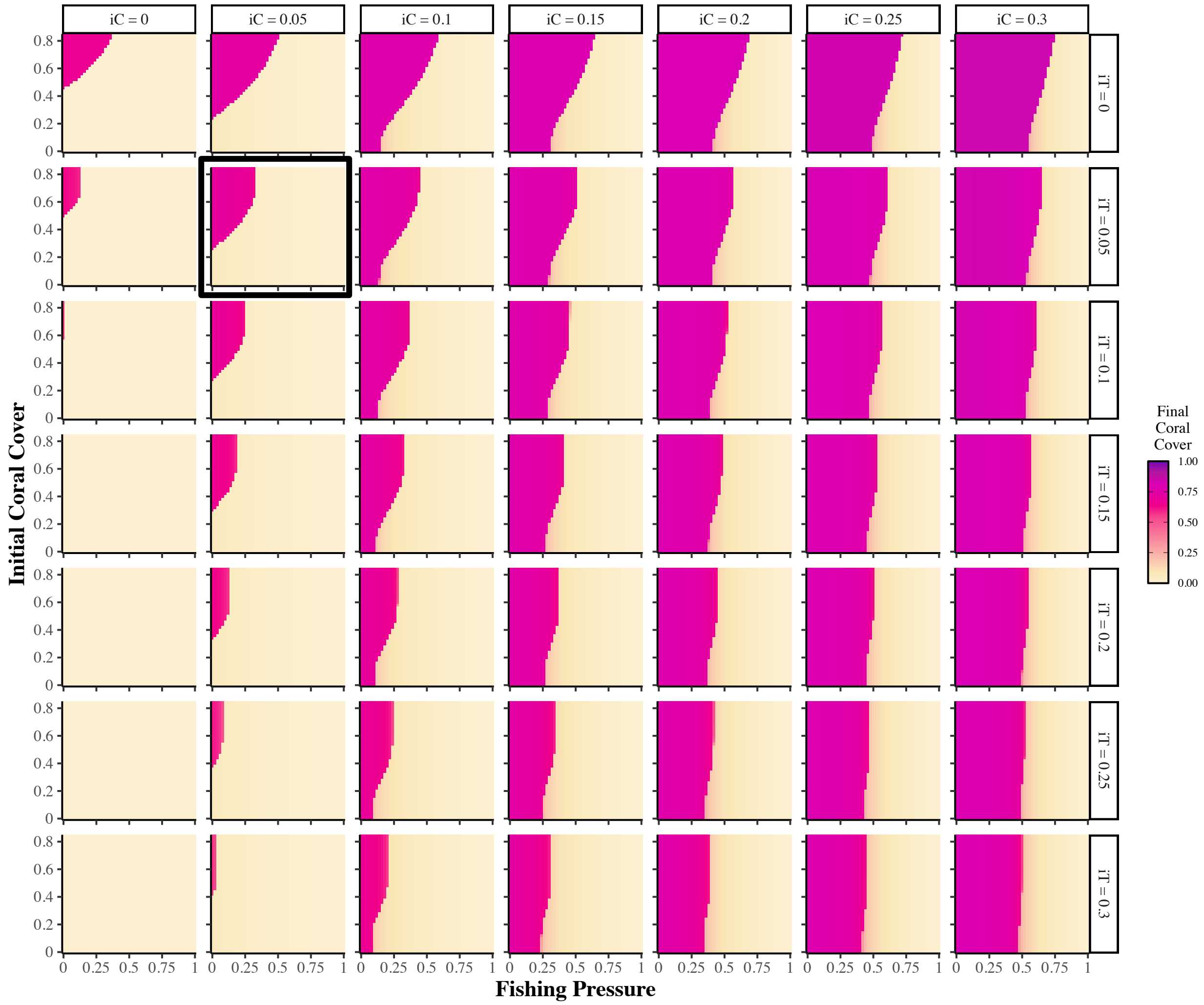

Figure X. Bistability sensitivity analysis for import of coral and turf propagules (parameters i_C and i_T) for grazer-dominated reefs. We examined i_C and i_T values ranging from 0 – 0.3 in increments of 0.05. The outlined panel indicates the parameter values we used in our model analyses ($i_C = 0.05$ and $i_T = 0.05$).

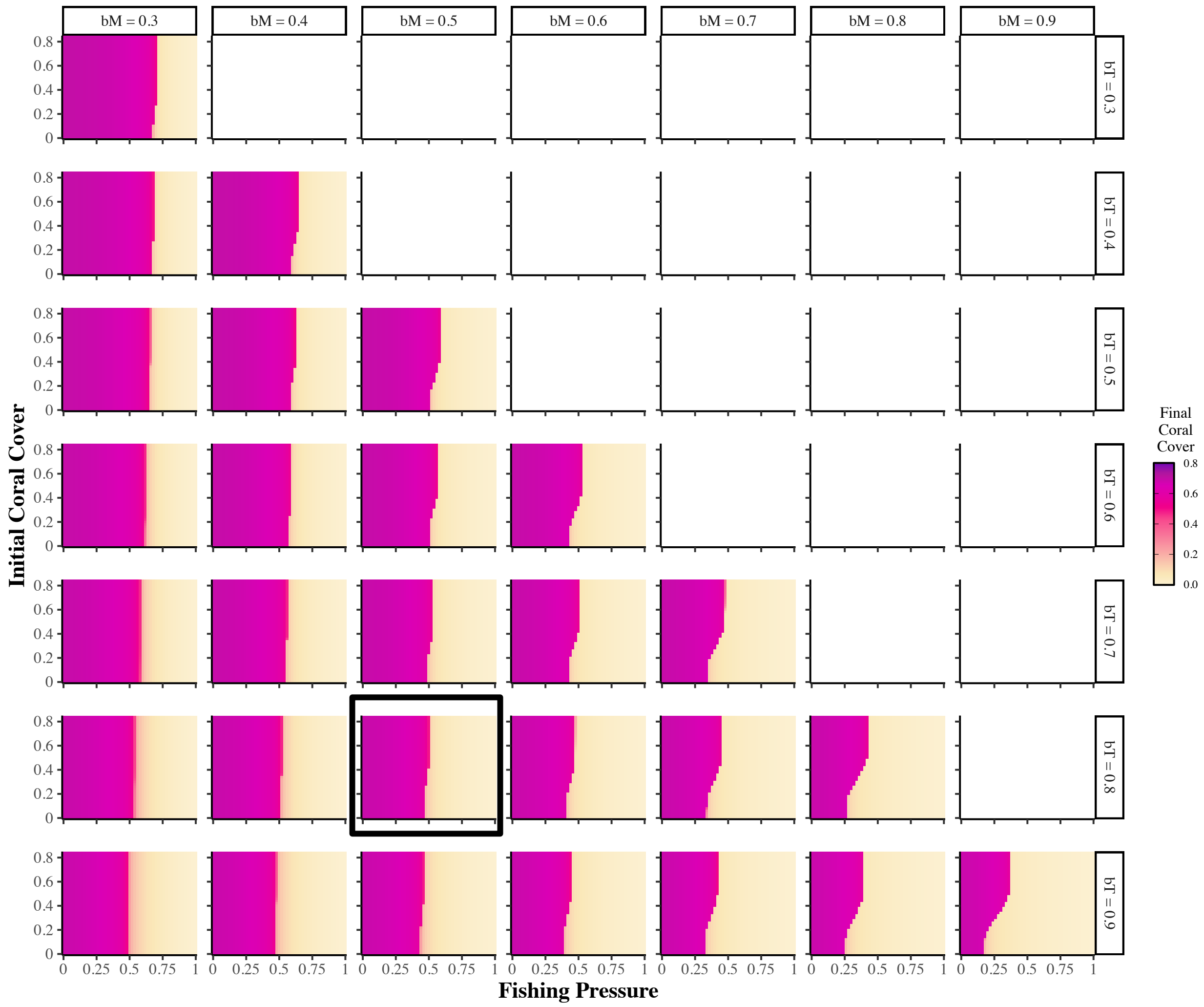

Figure Y. Bistability sensitivity analysis for expansion of turf and macroalgae (parameters b_T and b_M) for browser-dominated reefs. We examined b_T and b_M values ranging from 0.3 – 0.9 in increments of 0.1. The outlined panel indicates the parameter values we used in our model analyses ($b_T = 0.8$ and $b_M = 0.5$).

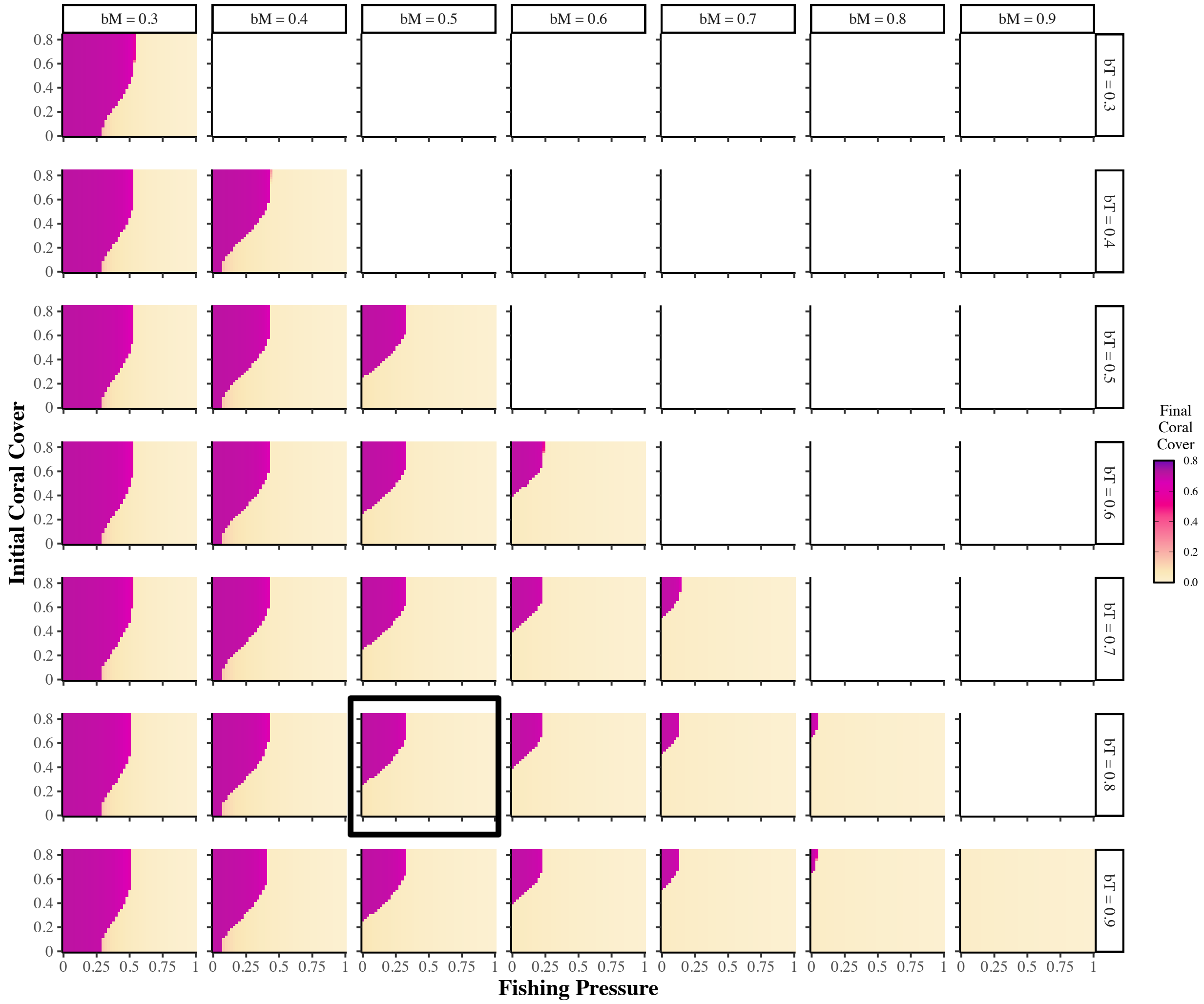

Figure Z. Bistability sensitivity analysis for expansion of turf and macroalgae (parameters b_T and b_M) for grazer-dominated reefs. We examined b_T and b_M values ranging from 0.3 – 0.9 in increments of 0.1. The outlined panel indicates the parameter values we used in our model analyses ($b_T = 0.8$ and $b_M = 0.5$).

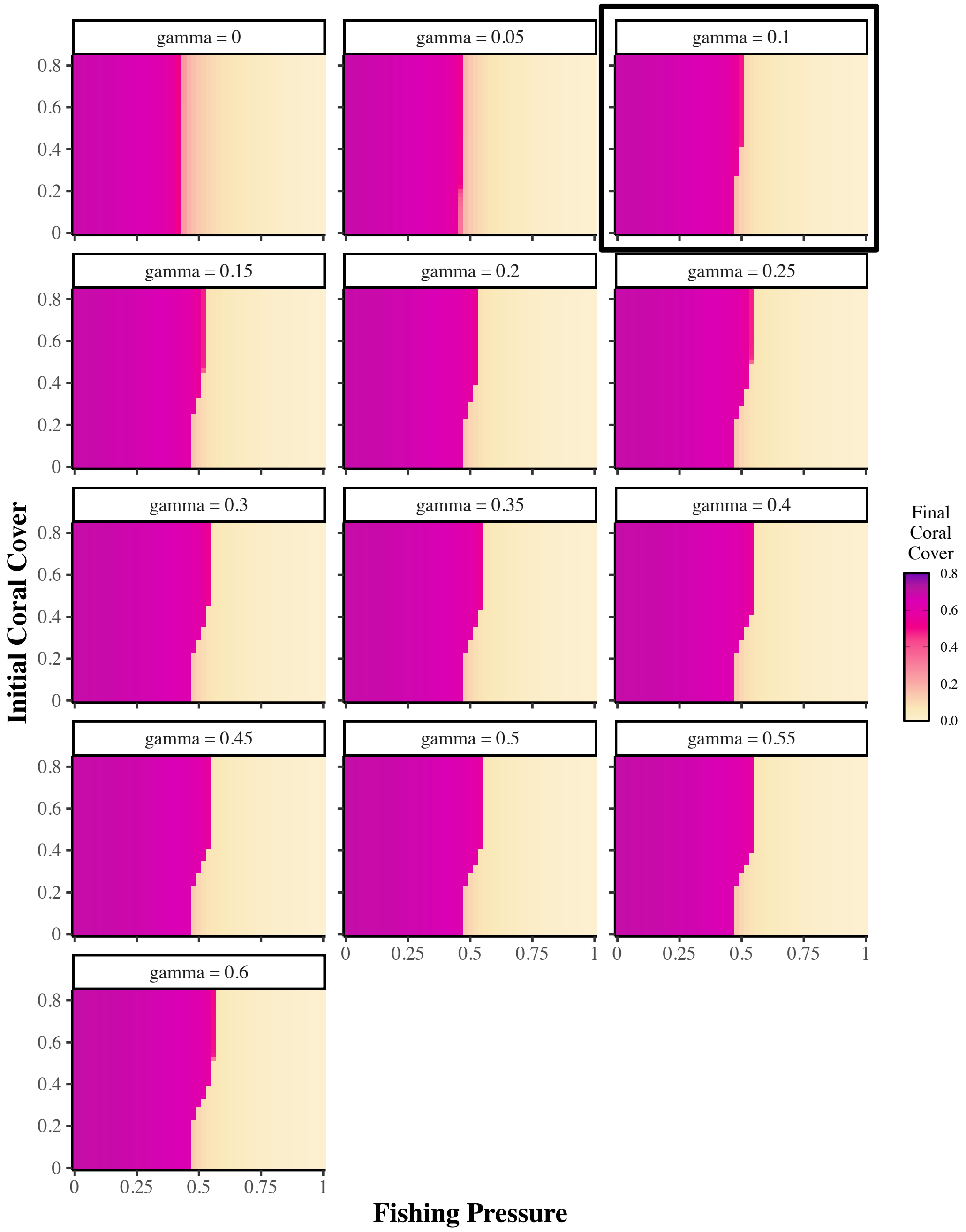

Figure AA. Bistability sensitivity analysis for transition from turf to macroalgae (parameter gamma γ) for browser-dominated reefs. We examined gamma (γ) values ranging from 0 – 0.6 in increments of 0.05. The outlined panel indicates the parameter value we used in our model analyses (γ = 0.1).

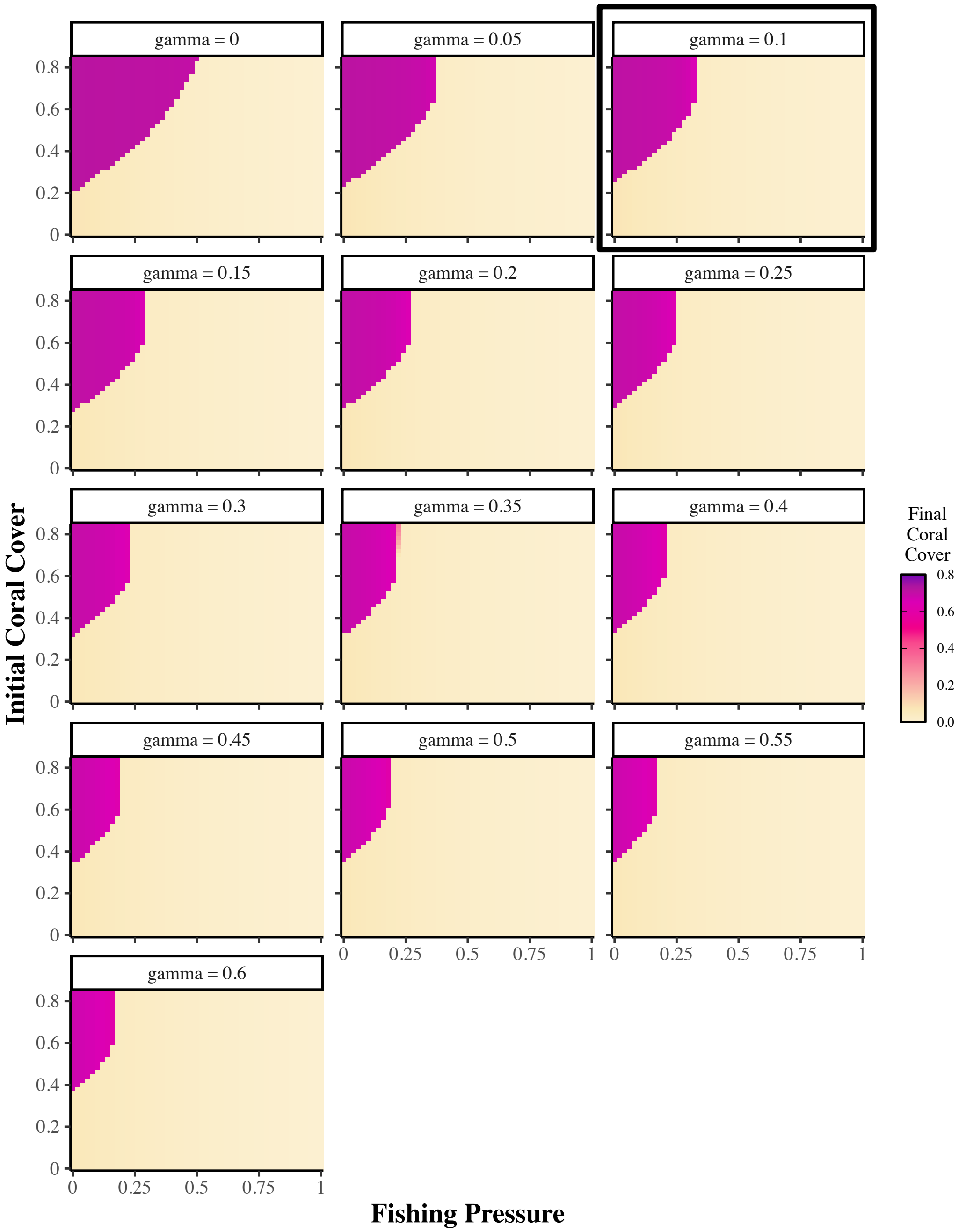

Figure BB. Bistability sensitivity analysis for transition from turf to macroalgae (parameter gamma γ) for grazer-dominated reefs. We examined gamma (γ) values ranging from 0 – 0.6 in increments of 0.05. The outlined panel indicates the parameter value we used in our model analyses (γ = 0.1).
